# HaloPROTAC3 does not trigger the degradation of the halotagged parasitophorous vacuole membrane protein UIS4 during *Plasmodium* liver stage development

**DOI:** 10.1038/s41598-025-98257-9

**Published:** 2025-05-26

**Authors:** Melanie Lam, Alexandra Probst, Laura Torres, Ashley A. Lantigua, Matthew E. Fishbaugher, Jyothsna R. Kumar, Manuel Saldivia, Allison Torres, Shreeya Hegde, Maya Aleshnick, Charlie Jennison, Sarah G. H. Roberson, Chester J. Joyner, Ashley M. Vaughan, Brandon K. Wilder, Carole Manneville, Erika L. Flannery, David Marcellin, Beat Nyfeler, Zacharias Thiel, Sebastian A. Mikolajczak, Anke Harupa, Gabriel Mitchell

**Affiliations:** 1 Open Innovation at Global Health Disease Area, Biomedical Research, Novartis, Emeryville, CA USA; 2 Global Health Disease Area, Biomedical Research, Novartis, Emeryville, CA USA; 3 Vaccine and Gene Therapy Institute, Oregon Health and Science University, Beaverton, OR USA; 4 Seattle Children’s Research Institute, Seattle, WA USA; 5 Department of Infectious Diseases, University of Georgia, Athens, GA USA; 6 Discovery Sciences, Biomedical Research, Novartis, Basel, Switzerland

**Keywords:** Targeted protein degradation, Induced proximity, Schizont, Hypnozoite, Infectious disease, Malaria, Cellular microbiology, Parasitology, Pathogens, Drug discovery, Malaria, Small molecules

## Abstract

Targeted protein degradation (TPD) is a novel strategy for developing therapeutics against pathogens. Prior to causing malaria, *Plasmodium* parasites replicate within hepatocytes as liver stages, surrounded by a parasitophorous vacuole membrane (PVM). We hypothesized that TPD can be employed to trigger host-driven degradation of essential liver stage PVM proteins and lead to parasite death. To explore this, we took advantage of the proteolysis-targeting-chimera HaloPROTAC3, a molecule that recruits the host von Hippel-Lindau (VHL) E3 ligase to the HaloTag (HT). Parasites expressing HT fused to the host cytosol-exposed domain of the PVM protein UIS4 (UIS4-HT) were generated in *Plasmodium berghei* and *Plasmodium cynomolgi*, but only *P. berghei* UIS4-HT enabled productive liver stage infection experiments in vitro. Although HaloPROTAC3 triggered the degradation of HT proteins in host cells, it had no impact on the survival of *P. berghei* UIS4-HT liver stages. Furthermore, HaloPROTAC3 bound to *P. berghei* UIS4-HT but did not recruit VHL or trigger ubiquitination of the PVM. Overall, although this study did not establish whether host-driven TPD can degrade *Plasmodium* PVM proteins, it highlights the challenges of developing TPD approaches against novel targets and offers insights for advancing this therapeutic strategy against pathogens.

**Supplementary Information:**

The online version contains supplementary material available at 10.1038/s41598-025-98257-9.

## Introduction

Despite the current arsenal of antimicrobials, infectious diseases continue to have a profound impact on global health^1^. Microbial pathogens exhibit a remarkable ability to adapt and withstand the effects of antimicrobials, either by evolving resistance mechanisms or by adopting strategies to survive harsh conditions^2^. The constant emergence of microbes that tolerate traditional antimicrobial treatments fuels the urgent need for therapeutics with novel mechanisms of action. Exploiting host processes involved in microbial pathogenesis or the host immune response represents an overlooked avenue for the development of therapeutics^3,4^. While host-directed therapeutics may face limitations in terms of potential adverse effects, they may complement microbe-targeted treatments, and their benefits might outweigh the risks associated with their development^3,4^.

Malaria is a widespread disease primarily caused by *Plasmodium falciparum* and *Plasmodium vivax* that affects hundreds of millions of individuals annually^5^. In addition to the threat posed by the development of resistance to currently available antimalarial therapeutics, a significant hurdle in the fight against malaria is the ability of *P. vivax* to enter a dormant state in the liver, which leads to clinical relapses even months after the initial infection and facilitates the spread of the disease^6^. Shortly after being introduced into the mammalian host through a mosquito bite, *Plasmodium* parasites infiltrate the liver and invade hepatocytes. Within a protected parasitophorous vacuole (PV), parasites then undergo thousands of replication cycles through a process called schizogony, prior to being released into the bloodstream and causing malaria^7^. In addition to replicative schizonts, *P. vivax* also forms dormant liver stage hypnozoites, which are tolerant to most antimalarial drugs and can develop into schizonts, leading to relapses^6^. The PV membrane (PVM), which separates both schizonts and hypnozoites from the host cytosol, contains essential *Plasmodium* PVM proteins such as some members of the early transcribed membrane protein (ETRAMP) family^8^. These proteins have their C-terminal region exposed to the host cytosol and are presumed to interact with hepatocyte processes although their exact functions remain elusive^9–12^. However, these PVM proteins are not typical targets for the development of drugs because of their usual lack of enzymatic activities and of known functional domains.

Progress in understanding the biology of *P. vivax* and other *Plasmodium* species causing relapsing malaria is hindered by significant technical obstacles^6,13^. *P. vivax* lacks a continuous blood culture system, which makes experiments mostly reliant on access to human samples and hinders the study of clonal lines and the development of transgenic tools. The simian parasite *Plasmodium cynomolgi* also forms liver stage hypnozoites and is thus a valuable surrogate model for studying relapsing malaria. More precisely, *P. cynomolgi* facilitates the generation of transgenic parasites and, because of the possibility to perform infections in non-human primates (NHPs), *P. cynomolgi* allows the reproducibility of experiments with specific strains and clonal lines. Nevertheless, conducting experiments to study *P. vivax* and *P. cynomolgi* liver stages is arduous and involves resource-intensive cellular assays with primary hepatocytes or in vivo infection models^6,13^. Due to these challenges, most of our insights on liver stage biology were gained from studying rodent malaria parasites (e.g., *Plasmodium berghei*), which rapidly develop liver stages in immortalized cell lines and are genetically tractable^7^. However, rodent *Plasmodium* parasites are inadequate for studying relapsing malaria since they do not form hypnozoites.

Novel therapeutic strategies are needed to tackle relapsing malaria due to the challenges associated with developing drugs that are effective against hypnozoites. Induced proximity represents a revolutionary approach to drug discovery that leverages the concept of bringing two molecules into close proximity to modulate their function, allowing the manipulation of molecular interactions and the co-option of several cellular processes (e.g., protein degradation and stabilization, the autophagy-lysosomal pathway, and post-translational modifications)^14^. Proximity-inducing therapeutics may offer several advantages over conventional therapeutics, including high selectivity and the ability to target a wide range of components such as traditionally undruggable proteins, nucleic acids, organelles, and even agents causing infectious diseases^14,15^. Induced proximity could be used to harness host processes and trigger the elimination of intracellular *Plasmodium* parasites, thus bypassing the slow metabolism and the inherent antimalarial tolerance exhibited by hypnozoites.

Targeted protein degradation (TPD) is a proximity-inducing approach that exploits the proteasomal degradation machinery to selectively eliminate specific proteins^16^. More specifically, TPD can be achieved using heterobifunctional proteolysis targeting chimeras (PROTACs) or molecular glues that induce proximity between a target protein and an E3 ubiquitin (Ub) ligase, resulting in polyubiquination of the target, which marks it for proteasomal degradation. While much of the TPD landscape remains unexplored^17^, several TPD-based drugs are already in the pipeline against cancer and are progressing through the different stages of development^16^. TPD is also gaining traction as a potential treatment modality for infectious diseases, including viral and other microbial infections^15^. Notably, the potential to degrade non-enzymatic and membrane-associated proteins^18,19^ makes TPD an appealing strategy for targeting *Plasmodium* PVM proteins and for developing drugs with activity against *P. vivax* hypnozoites.

**Fig. 1:**
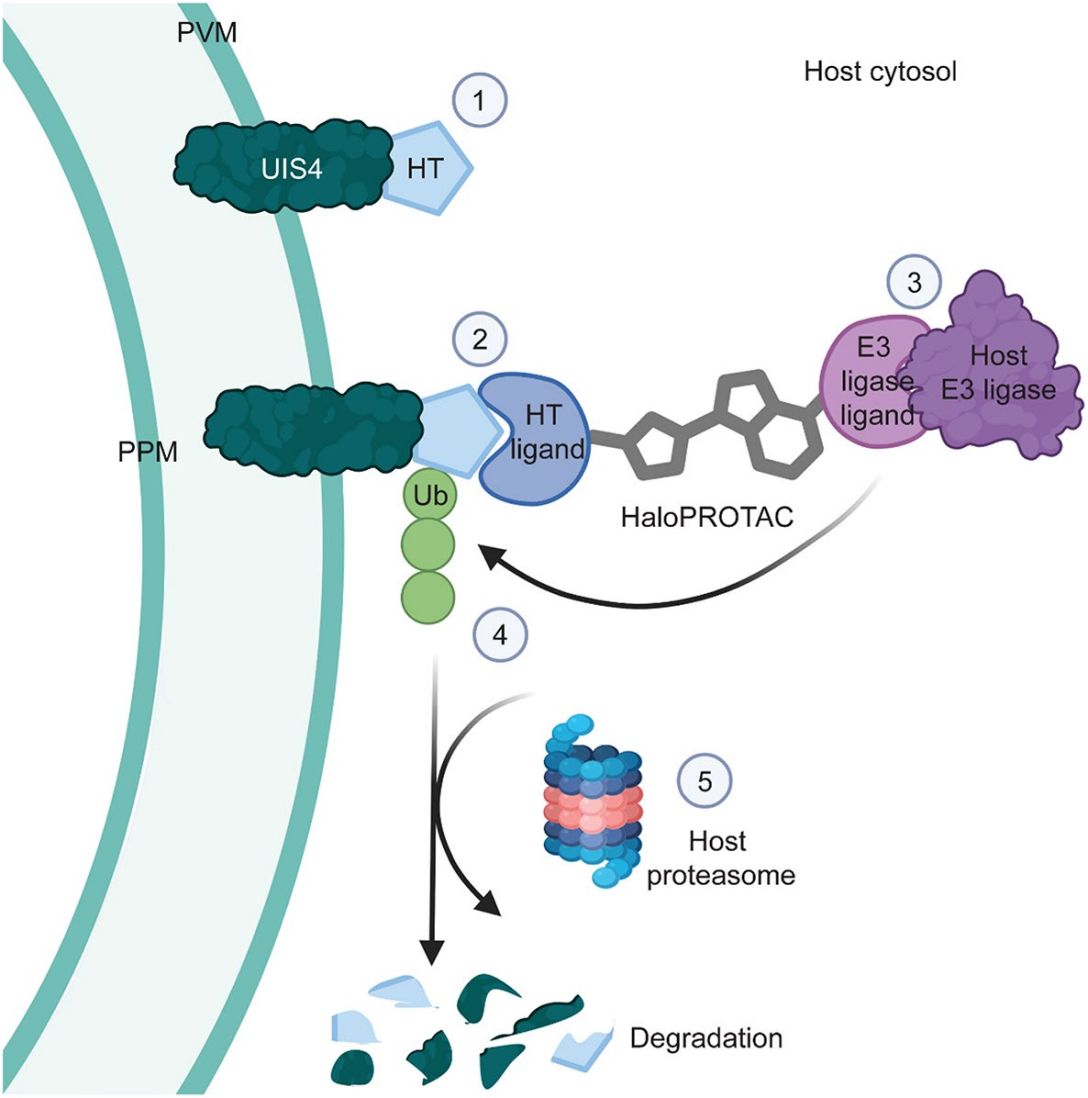
Host-driven targeted degradation of a *Plasmodium* liver stage PVM protein using HaloTag technology. The illustration shows: **(1)** the host cytosol-exposed C-terminus of the PVM protein UIS4 fused to HaloTag (HT), **(2)** the binding of the reactive chloroalkane HaloTag ligand (HTL) moiety of a HaloPROTAC to the UIS4-HT fusion protein, **(3)** the binding of a host E3 ligase to the E3 ligase ligand moiety on the same HaloPROTAC, and **(4)** the ubiquitination and **(5)** the proteasomal degradation of UIS4-HT. PVM, parasitophorous vacuole membrane; PPM, parasite plasma membrane; Ub, ubiquitin; HaloPROTAC, HaloTag-targeted proteolysis targeting chimeras.

This study aimed to investigate the potential utilization of host-driven TPD against *Plasmodium* PVM proteins expressed by liver stage schizonts and hypnozoites (Fig. 1). As proof of concept, we used a heterobifunctional compound (HaloPROTAC3) that binds the self-labeling protein HaloTag (HT) as well as the von Hippel-Lindau (VHL) E3 ligase^20^. More specifically, *P. berghei* and *P. cynomolgi* transgenic parasites expressing a HaloTagged version of the PVM protein upregulated in infectious sporozoites 4 (UIS4)^21^ were generated with the objective of using them in combination with HaloPROTAC3 to study host-driven TPD of a liver stage PVM protein.

## Results

### Generation and characterization of *Plasmodium* parasites expressing UIS4-HT

**Fig. 2:**
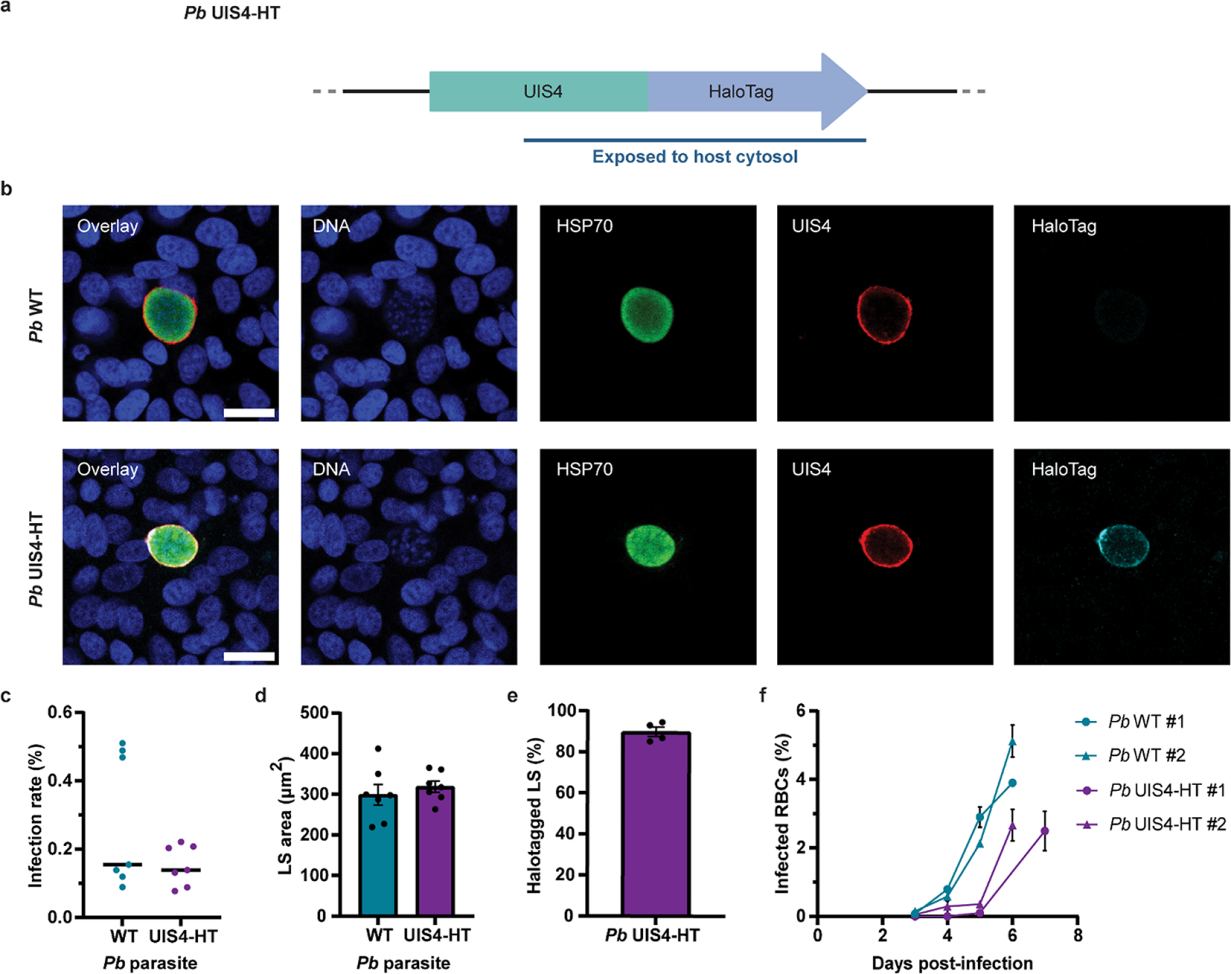
Generation and characterization of *P. berghei* (*Pb*) UIS4-HT. (**a**) Illustration of the *P. berghei* UIS4-HT locus. The blue line indicates the portion that is exposed to the host cytosol. (**b**) Confocal micrographs of Huh7 cells infected with *P. berghei* WT and *P. berghei* UIS4-HT for 2 days and stained for DNA (blue), *Pb* HSP70 (green), *Pb* UIS4 (red), and HaloTag (cyan). Scale bars are 20 μm. (**c**) Infection rates (% liver stages (LS) per host nuclei). Median infection rates were calculated from 2–4 technical replicates and pooled from 7 independent experiments (*N* = 7). No statistically significant difference was detected (*P* = 0.32, Mann-Whitney test). The solid black lines indicate medians. (**d**) Area sizes of liver stages at 2 days post-infection. Median area sizes were determined for each well and averaged from 2–4 technical replicates. No statistically significant difference was detected (*P* = 0.51, unpaired *t* test, *N* = 7). Results are expressed as means with SEMs. (**e**) Proportion of HaloTag-positive liver stages for Huh7 cells infected with *P. berghei* UIS4-HT. Median values were calculated from 2–3 technical replicates and expressed as means with SEMs (*N* = 4). (**f**) Blood stage parasitemia in mice infected with *P. berghei* WT and *P. berghei* UIS4-HT sporozoites. The graph shows data from two independent experiments, each including 2 (#1) or 3 (#2) mice per condition apart from one mouse for *P. berghei* WT #1 at 6 days post-infection (one mouse had to be euthanized a day earlier for this group). Results are expressed as means with SEMs. RBCs, red blood cells.

To enable proof-of-concept studies using HaloPROTAC3 against a liver stage *Plasmodium* PVM protein, a transgenic *P. berghei* parasite was created by fusing HT to the host cytosol-exposed C-terminus of UIS4^12,21^ (Fig. 2a) using a previously described allelic replacement strategy^22^. The resulting transgenic population was validated using PCR genotyping (Supplementary Fig.  S1) and characterized with a microscopy-based liver stage infection assay in the Huh7 human hepatoma cell line (Fig. 2b–e). The transgenic parasites tend to form fewer liver stages compared to wild-type *P. berghei* (not statistically significant, Fig. 2c), demonstrated proper liver stage development (Fig. 2d) and expressed the HaloTagged UIS4 at the PVM (Fig. 2b and e). Furthermore, mice inoculated with UIS4-HT salivary gland sporozoites established blood stage infection, albeit with delay compared to wild-type parasites, confirming the ability of transgenic parasites to complete the *Plasmodium* life cycle (Fig. 2f). Overall, these findings demonstrated that the transgenic *P. berghei* parasites are virulent and express UIS4-HT at the PVM during the liver phase of infection.

**Fig. 3:**
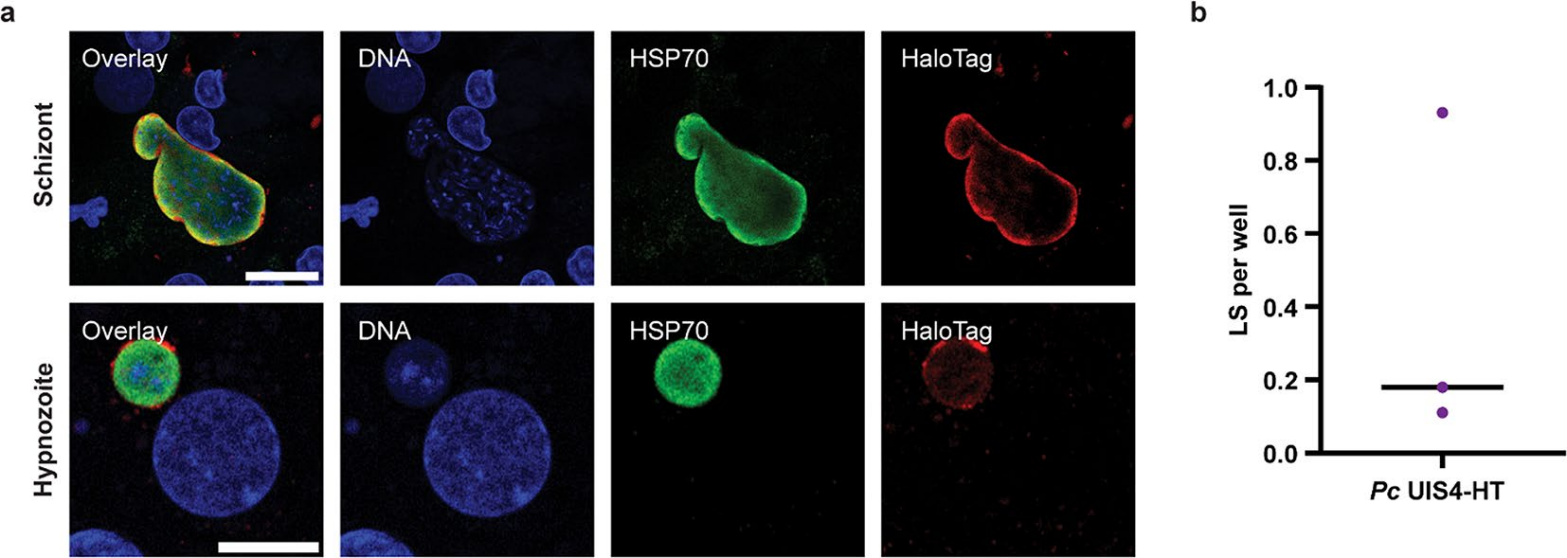
Characterization of *P. cynomolgi* (*Pc*) UIS4-HT liver stages. (**a**) Airyscan micrographs of primary simian hepatocytes infected with *P. cynomolgi* UIS4-HT for 8 days and stained for DNA (blue), *Pc* HSP70 (green), and HaloTag (red). A schizont (top row) and a hypnozoite (bottom row) are shown. Scale bars are 20 μm and 10 μm, respectively. (**b**) Infection rate of *P. cynomolgi* UIS4-HT presented as number of liver stages (LS) per well (≥ 80 wells per biological replicate). The solid black line indicates the median (*N* = 3).

In an attempt to investigate TPD during the liver phase of relapsing malaria, we generated transgenic *P. cynomolgi* UIS4-HT parasites in vitro using a culture-adapted Berok strain and Cas9 technology^23–25^. Following genome editing, blood stage UIS4-HT parasites were validated using genotyping (Supplementary Fig. S2) and inoculated into non-human primates (NHPs). Blood from infected animals was then used to feed mosquitoes and produce sporozoites. Inoculation of UIS4-HT sporozoites into a naïve NHP resulted in a blood stage infection 12 days later (Supplementary Fig. S2), confirming the ability of the transgenic parasites to complete the *P. cynomolgi* life cycle. Moreover, a relapse was observed on day 42 post-infection following treatment of the primary infection with the blood stage drug Coartem (Supplementary Fig. S2), suggesting that *P. cynomolgi* UIS4-HT can form hypnozoites in vivo. Accordingly, in vitro infection of primary NHP hepatocytes with salivary gland transgenic sporozoites led to the formation of HaloTagged schizonts and hypnozoites (Fig. 3a). However, the infection rate associated with *P. cynomolgi* UIS4-HT was extremely low (i.e., between 0.11 and 0.93 liver stages per well seeded with 2.2 × 10^4^ hepatocytes) (Fig. 3b). Therefore, it was concluded that *P. cynomolgi* UIS4-HT is unsuitable for conducting more complex liver stage infection experiments, and the focus of the study was shifted towards gaining insights solely using *P. berghei* UIS4-HT.

### Validation of HaloPROTAC3 using cell-based assays

**Fig. 4:**
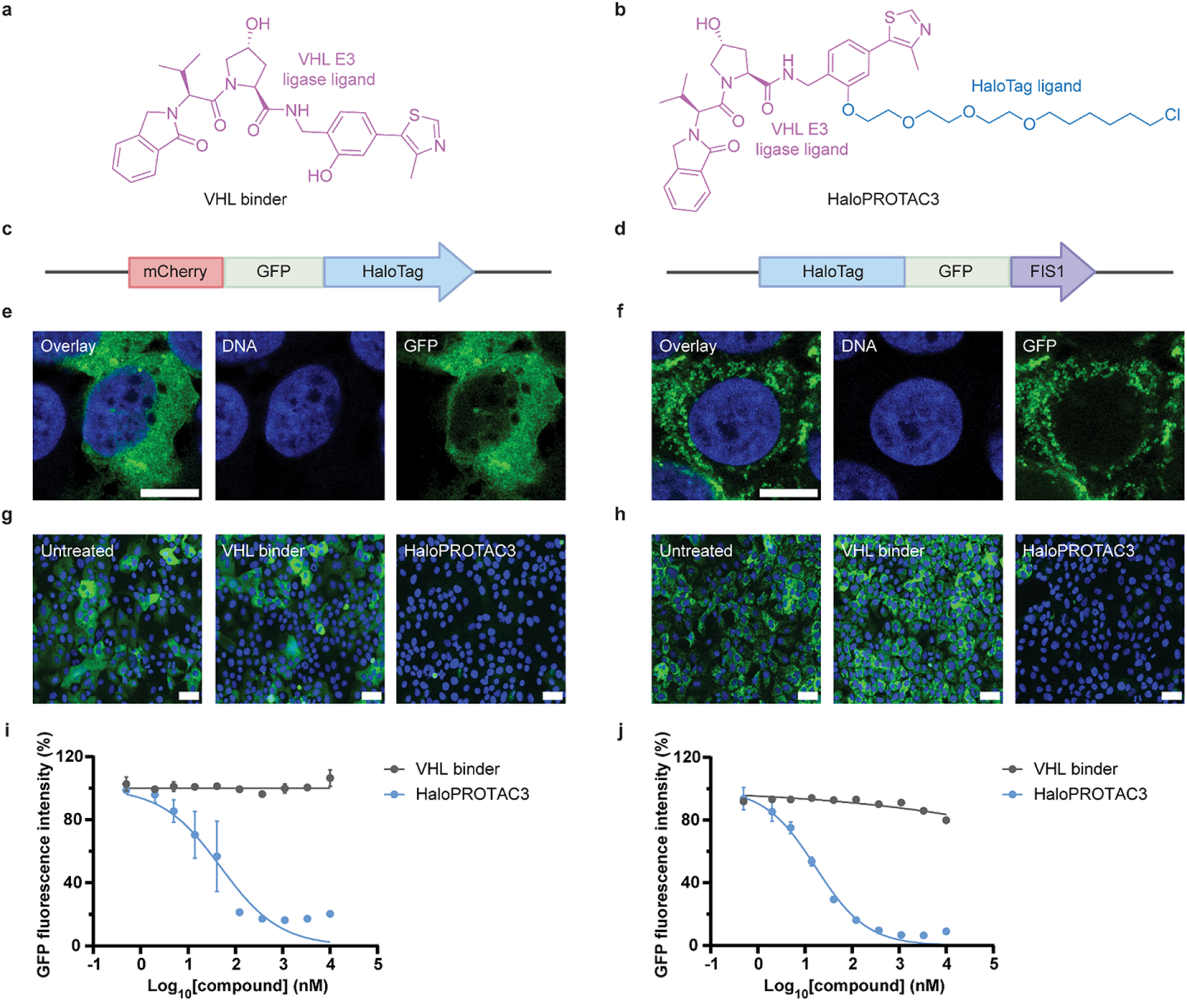
VHL-recruiting HaloPROTAC3 triggers the degradation of soluble cytosolic and transmembrane HT fusion proteins in Huh7 cells. Chemical structure of **(a)** the VHL control binder and **(b)** HaloPROTAC3. Illustration of the **(c)** mCherry-GFP-HT and **(d)** HT-GFP-FIS1 constructs expressed in Huh7 cells. Airyscan micrographs showing DNA (blue) and GFP fluorescence (green) in **(e)** mCherry-GFP-HT and **(f)** HT-GFP-FIS1 Huh7 cells. Scale bars are 10 μm. Confocal micrographs of Huh7 **(g)** mCherry-GFP-HT and **(h)** HT-GFP-FIS1 cells treated with 1 µM of the VHL binder or HaloPROTAC3. Scale bars are 50 μm. Dose-response curves showing that HaloPROTAC3 triggers degradation of **(i)** soluble cytosolic mCherry-GFP-HT and **(j)** transmembrane HT-GFP-FIS1 proteins in Huh7 cells. Average fluorescence intensities from technical duplicates were normalized and presented as means with SEMs (*N* = 2–3). 100% corresponds to DMSO-treated samples. GFP, green fluorescent protein; FIS1, mitochondrial fission 1 protein.

Before proceeding with further *P. berghei* UIS4-HT infection experiments, validation assays were conducted to assess the activity of a VHL control binder (without the HaloTag ligand portion) (Fig. 4a) and HaloPROTAC3^20^ (Fig. 4b) in relevant host cells. To evaluate the activity of these compounds against soluble and transmembrane proteins, the latter of which provides a better model for the PVM protein UIS4, Huh7 cells expressing HaloTagged fluorescent fusion proteins that localized within the host cytosol (mCherry-GFP-HT, Fig. 4c) or at the outer mitochondrial membrane (HT-GFP-FIS1, Fig. 4d) were generated by adapting previously described approaches^20,26,27^. The subcellular localization of each fusion protein was confirmed via microscopy analysis to be cytosolic (i.e., diffuse distribution pattern; Fig. 4e) or mitochondrial (i.e., network-like distribution pattern colocalizing with MitoTracker; Fig. 4f and Supplementary Fig. S3), respectively. Interestingly, HaloPROTAC3 demonstrated comparable degradation capacity for both mCherry-GFP-HT (Fig. 4g) and HT-GFP-FIS1 (Fig. 4h), with 50% maximum activity concentrations in the nanomolar range (Fig. 4i-j). It was also confirmed that human primary hepatocytes, the most relevant cells for studying the liver stages of *Plasmodium* parasites causing malaria in humans, degrade the soluble cytosolic reporter GFP-HT when exposed to HaloPROTAC3 (Supplementary Fig. S4). Moreover, we confirmed that HaloPROTAC3 triggers degradation of the HaloTagged C-terminal region of *P. berghei* UIS4, which corresponds to the host cytosol-exposed domain, when recombinantly expressed in Huh7 cells (Supplementary Fig. S5). These findings validate the ability of HaloPROTAC3 to induce the degradation of HaloTagged proteins in both Huh7 cells and primary hepatocytes.

### HaloPROTAC3 does not impact *P. berghei* UIS4-HT liver stages

**Fig. 5:**
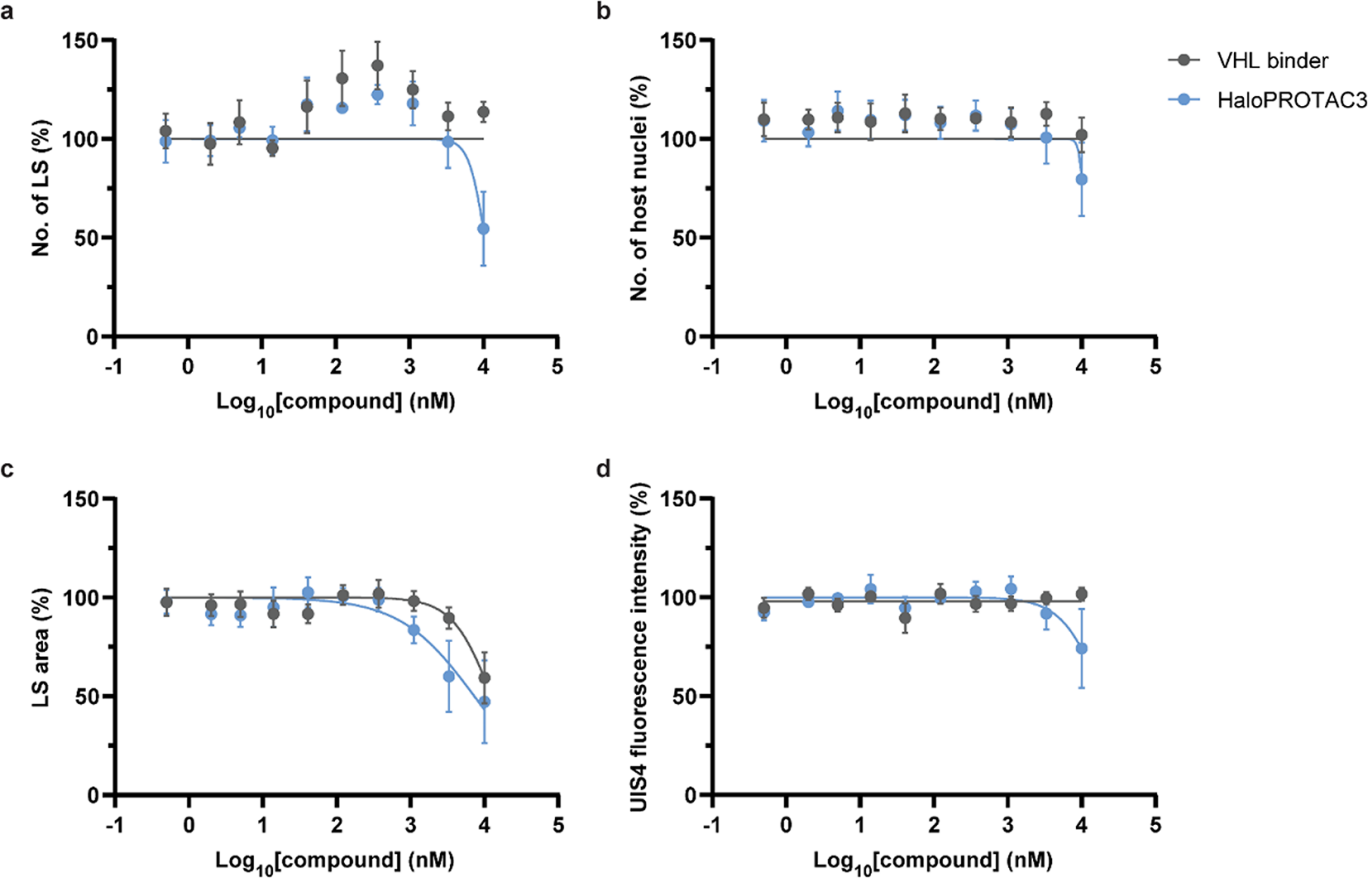
HaloPROTAC3 does not influence the development of *P. berghei* UIS4-HT liver stages (LS). The impact of the VHL binder and HaloPROTAC3 on the **(a)** number of *P. berghei* liver stages and **(b)** number of host nuclei was determined in Huh7 cells 2 days post-infection. Results were normalized and median values were calculated from 2–3 technical replicates. Results are presented as means with SEMs (*N* = 4). The impact of compounds on **(c)** liver stage sizes and **(d)** the median UIS4 fluorescence intensity of *P. berghei* liver stages was also determined. Median area sizes and medians of median UIS4 fluorescence intensities of *P. berghei* liver stages were calculated for each well, normalized, and averaged from 2–3 technical replicates. 100% corresponds to infected samples treated with DMSO. Results are presented as means with SEMs (*N* = 4). Statistically significant differences between the VHL binder and HaloPROTAC3 were detected only for the number of *P. berghei* liver stages and UIS4 intensities at 10,000 nM of compounds (*P* < 0.05, two-way ANOVA with Bonferroni post-hoc test).

The impact of HaloPROTAC3 on *P. berghei* UIS4-HT was assessed in Huh7 cells. Specifically, host cells were infected with salivary gland sporozoites, treated with a range of compound concentrations, and the formation of liver stages was evaluated 2 days post-infection using immunofluorescence and high-content imaging (Fig. 5). HaloPROTAC3 did not affect the number of UIS4-HT liver stages, except for a decrease at 10,000 nM of compounds, which was attributed to cytotoxicity, as suggested by the concomitant decrease in host nuclei counts (Fig. 5a-b). Moreover, it was observed that HaloPROTAC3 did not specifically impact the size of liver stages, as both the control VHL binder and HaloPROTAC3 produced similar effects (Fig. 5c). To more directly monitor the level of UIS4 expression at the PVM of *P. berghei* liver stages, the fluorescence intensity of immunostained UIS4 was measured during liver stage infection but was unaffected by exposure to compounds (except at 10,000 nM) (Fig. 5d).While it is possible that slight differences in PVM protein levels are below the detection limit of our assay, the findings indicate that HaloPROTAC3 does not effectively degrade UIS4-HT. Overall, these results suggest that HaloPROTAC3 does not impact the development of UIS4-HT *P. berghei* liver stages.

### HaloPROTAC3 binds to *P. berghei* UIS4-HT liver stages

**Fig. 6:**
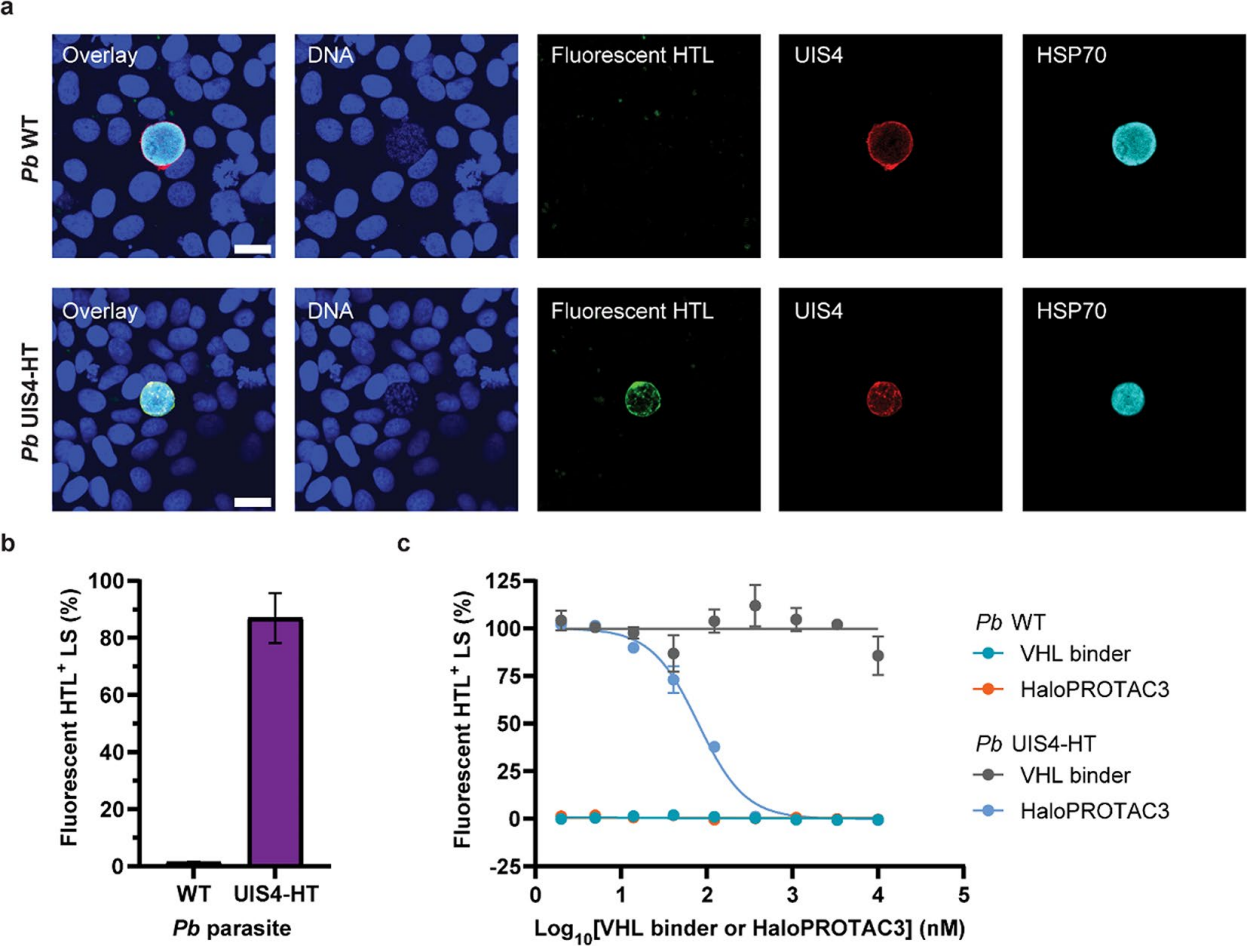
HaloPROTAC3 binds to *P. berghei* (*Pb*) UIS4-HT liver stages (LS). **(a)** Confocal micrographs of Huh7 cells infected with *P. berghei* WT and *P. berghei* UIS4-HT and stained for DNA (blue), parasite markers UIS4 (red) and HSP70 (cyan), and a fluorescent HaloTag ligand (HTL, green) 2 days post-infection. Scale bars are 20 μm. **(b)** Percentages of liver stages with bound fluorescent HTL were quantified for cells infected with *P. berghei* WT and *P. berghei* UIS4-HT. Median percentages were calculated from 4–8 technical replicates and expressed as means with SEMs (*N* = 2). **(c)** Dose-response curves showing the percentages of liver stages that bound to the fluorescent HTL as a function of HaloPROTAC3 concentration. During this assay, the binding of HaloPROTAC3 to UIS4-HT liver stages was evaluated by probing for remaining available binding sites with the fluorescent HTL. *P. berghei* WT and the VHL binder served as negative controls. Median percentages of liver stages with bound fluorescent HTL were calculated from technical duplicates and normalized. 100% corresponds to samples infected with *P. berghei* UIS4-HT and treated with DMSO. Results are means with SEMs (*N* = 2).

To gain insights into why HaloPROTAC3 induces degradation of soluble and transmembrane host proteins (Fig. 4 and Supplementary Fig. S4-S5), but not UIS4 at the *Plasmodium* PVM (Fig. 5), we investigated the binding of HaloTag ligands (HTLs) to UIS4-HT liver stages. We first showed that the addition of a fluorophore-conjugated HTL (hereafter referred to as fluorescent HTL) (Supplementary Fig. S6) to infected cells resulted in the specific staining of *P. berghei* UIS4-HT liver stages (Fig. 6a-b). To confirm binding of HaloPROTAC3 to transgenic liver stages, *P. berghei*-infected cells were treated with various concentrations of HaloPROTAC3 followed by treatment with the fluorescent HTL to probe for remaining available binding sites^28^. The results confirmed specific, concentration-dependent reduction of the number of HTL-positive UIS4-HT liver stages by HaloPROTAC3 (Fig. 6c). Thus, these findings confirm that HaloPROTAC3 can bind to UIS4-HT liver stages.

### HaloPROTAC3 does not induce VHL recruitment and PVM ubiquitination during *P. berghei* liver stage development

**Fig. 7:**
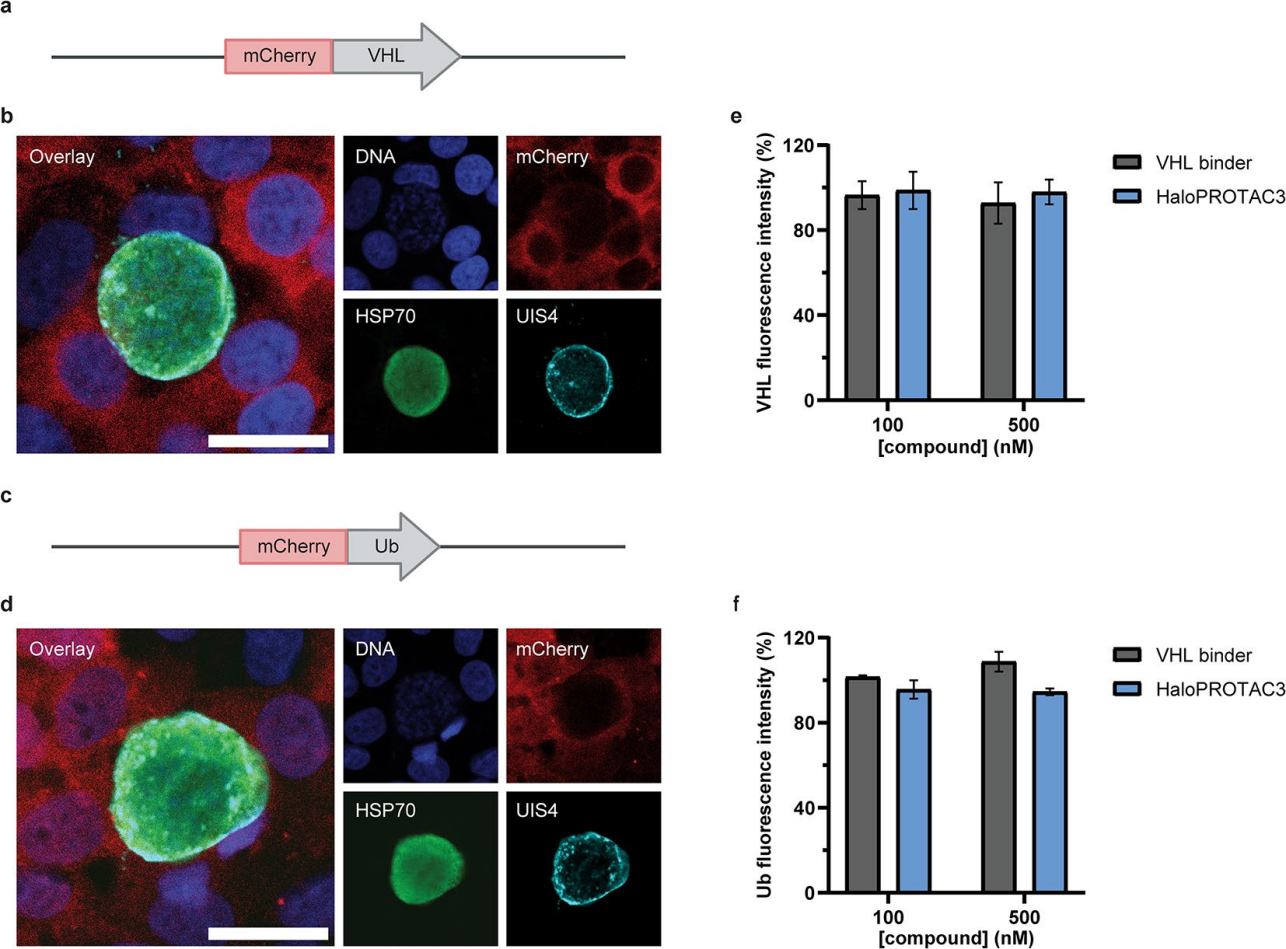
HaloPROTAC3 does not induce VHL recruitment and PVM ubiquitination during *P. berghei* liver stage development. **(a)** Illustration of the mCherry-VHL construct expressed in Huh7 cells. **(b)** Confocal micrographs of Huh7 mCherry-VHL cells infected with *P. berghei* UIS4-HT and stained for DNA (blue), mCherry (red), and parasite markers HSP70 (green) and UIS4 (cyan). Scale bar is 20 μm. **(c)** Illustration of the mCherry-ubiquitin (Ub) construct expressed in Huh7 cells. **(d)** Confocal micrographs of Huh7 mCherry-Ub cells infected with *P. berghei* UIS4-HT and stained for DNA (blue), mCherry (red), and parasite markers HSP70 (green) and UIS4 (cyan). Scale bar is 20 μm. Integrated mCherry-VHL **(e)** and mCherry-Ub **(f)** fluorescence intensities associated with *P. berghei* UIS4-HT liver stages in Huh7 cells treated with either the VHL binder or HaloPROTAC3. The median integrated fluorescence intensities of *P. berghei* liver stages were calculated for each well, normalized and averaged from 4 technical replicates. 100% corresponds to infected samples treated with DMSO. Results are presented as means with SEMs (*N* = 2). No statistically significant differences were detected at 100 or 500 nM (unpaired *t* tests).

To assess if treatment with HaloPROTAC3 leads to the recruitment of VHL to UIS4-HT, followed by ubiquitination of the PVM, Huh7 reporter cells expressing mCherry-VHL (Fig. 7a-b) and mCherry-ubiquitin (Ub) (Fig. 7c-d) were generated and used to monitor protein localization during *P. berghei* infection. It is worth noting that while previous studies similarly used N-terminal tagging of ubiquitin with a fluorescent protein to study *P. berghei* liver stages^29,30^, we are not aware of any studies confirming the feasibility of this approach to assess VHL recruitment to the PVM. Interestingly, the mCherry-VHL and mCherry-Ub fusion proteins did not undergo any re-localization upon exposure to 100 and 500 nM of HaloPROTAC3 (Fig. 7e-f), concentrations at which binding to UIS4-HT was previously observed (Fig. 6c). These results suggest that VHL and ubiquitin were not recruited to UIS4-HT liver stages following HaloPROTAC3 treatment. However, it is also possible that the association of VHL and ubiquitin with the UIS4-HT PVM was only transient and not detectable using this approach.

It was then hypothesized that the recruitment of VHL to UIS4-HT liver stages is obstructed by steric hindrance at the PVM. The position and orientation of bound HaloPROTAC3 in relation to the PVM membrane and other PVM proteins might indeed impede the recruitment of VHL to UIS4-HT, thereby affecting the ubiquitination of the PVM. To evaluate this hypothesis, cell lines expressing SNAP-tag-GFP were generated and utilized with the bifunctional HaXS8 compound, which covalently dimerizes SNAP-tag and HaloTag^27,31^ (Supplementary Fig. S7) and should lead to long-lasting labelling of HaloTagged subcellular structures. Accordingly, HaXS8 treatment induced the re-localization of SNAP-tag-GFP to the mitochondrial network in cells expressing HT-mCherry-FIS1, thus validating this method to study the recruitment of host effectors to membrane-bound HaloTagged targets. However, HaXS8 did not significantly induce the recruitment of SNAP-tag-GFP to the PVM of UIS4-HT liver stages. Collectively, these results confirm that HaloPROTAC3 and HaXS8 are inefficient at inducing the recruitment of host proteins (i.e., VHL and SNAP-tag-GFP) to the PVM of UIS4-HT liver stages.

## Discussion

This study assessed host-driven TPD as a modality for degrading essential *Plasmodium* PVM proteins during *Plasmodium* liver stage development. To bypass the need for a *Plasmodium*-specific binder, proof-of-concept studies were conducted using HaloTag fusion proteins and HaloPROTAC3 (Fig. 1). Specifically, *P. berghei* and *P. cynomolgi* transgenic parasites expressing HaloTag fused with the host cytosol-exposed domain of the PVM protein UIS4 (UIS4-HT) were generated and characterized (Figs. 2 and 3 and Supplementary Fig. S1-S2). In contrast to *P. berghei*, *P. cynomolgi* UIS4-HT was unsuitable for performing further liver stage infection experiments because of its prohibitive infection rate (Fig. 3). Although HaloPROTAC3 triggered the degradation of soluble cytosolic and transmembrane HT fusion proteins ectopically expressed in host cells (Fig. 4 and Supplementary Fig. S4-S5), it had no effect on *P. berghei* UIS4-HT liver stages (Fig. 5). Moreover, while HaloPROTAC3 bound to UIS4-HT on the PVM of liver stages (Fig. 6), it failed to recruit VHL and trigger ubiquitination (Fig. 7). Overall, our results suggest that HaloPROTAC3 cannot induce the formation of a ternary complex with VHL and the transgenic *P. berghei* PVM protein UIS4-HT.

Transgenic parasites expressing UIS4-HT can transition through the life cycle but have a reduced infectivity in hepatocytes, which was more pronounced for *P. cynomolgi* UIS4-HT (Figs. 2 and 3). One hypothesis is that tagging UIS4 interferes with its essential function during liver stage development^21^. However, it is worth noting that *P. cynomolgi* UIS4-HT was generated using a culture-adapted Berok strain, which may have a reduced infectivity for hepatocytes compared to the *P. cynomolgi* M strain^24^. As such, it is likely that both the parasite background and the tagging of UIS4 contributed to the decreased infectivity of *P. cynomolgi* UIS4-HT. In addition to developing genetic tools in *P. vivax*^32,33^, future research comparing transgenic parasites generated in vitro (e.g., using culture-adapted Berok) and in vivo (e.g., using the M strain)^13^ could help optimizing approaches for the investigation of liver stage schizonts and hypnozoites in relapsing malaria species.

This study confirms that HaloPROTAC3 can induce the degradation of HT fusion proteins in the human hepatoma Huh7 cell line (Fig. 4 and Supplementary Fig. S5) and human primary hepatocytes (Supplementary Fig. S4), demonstrating that the host E3 ligase VHL can be harnessed for targeting the liver stages of human *Plasmodium* species. As the TPD field continues to evolve^17^, it will be crucial to explore the activity of other E3 ligases in hepatocytes and fully leverage the available chemical space for developing *Plasmodium*-specific small-molecule degraders.

Our findings indicate that HaloPROTAC3 binds to UIS4-HT at the PVM of *P. berghei* liver stages but fails to induce the formation of a ternary complex with VHL (Figs. 5, 6 and 7 and Supplementary Fig. S7). We speculate that either the orientation of the HaloTag, the linker length, or the presence of the PVM and other PVM proteins results in a geometry of the bound HaloPROTAC3 that is unfavorable for the formation of a ternary complex. An intriguing hypothesis is that UIS4, like other ETRAMPs^34^, forms complexes of oligomeric arrays in the PVM that prevent VHL recruitment. As growing *Plasmodium* liver stages have been shown to modulate the composition of their PVM through a membrane shedding process^35^, another possibility is that ternary complexes are being removed from the PVM. Alternatively, liver stages may shield their surface from certain host proteins, including E3 ligases, as observed for other intracellular microbial pathogens^36,37^. Future efforts should prioritize gaining a deeper understanding of the architecture and biology of the PVM, as this knowledge will facilitate the development of novel strategies for studying TPD during the liver phase of *Plasmodium* infection.

It is also worth noting that the inability of HaloPROTAC3 to induce the formation of a ternary complex at the PVM of liver stages may be specific to *P. berghei* UIS4-HT. Therefore, tagging the variable C-terminal domain of UIS4 from other *Plasmodium* species or of other liver stage PVM proteins (e.g., UIS3 or EXP1)^9,10,38^ with HaloTag could yield different results. Tagging *Plasmodium* proteins that are secreted into the host cell could also yield more favorable results (e.g., by being more amenable to TPD). An example of such *Plasmodium* proteins is the liver-specific protein 2 (LISP2), which translocates into the host nucleus after its secretion in the host cell, presumably affecting gene expression in hepatocytes^39,40^. Furthermore, HaloPROTACs with different linker lengths and geometries might better accommodate the formation of a ternary complex with UIS4-HT^20,41^. Incorporating different protein linkers^42^ between UIS4 and HaloTag, or exploring alternative tag systems^43–47^, might also facilitate the formation of ternary complexes. Finally, harnessing advancements in structural and computational biology for the rational design of compounds will facilitate the development of bifunctional binders against difficult targets^41,48,49^.

Besides TPD, the HaloTag system offers a wide array of applications for studying *Plasmodium* biology^50^. Due to the rapid and irreversible interaction of the HaloTag with HaloTag ligands, this system enables the efficient pulldown of molecular complexes and the investigation of protein-protein interactions^51^, which will aid in identifying partners for UIS4 and other PVM proteins. Additionally, the range of imaging tools utilizing the HaloTag^50^ could be applied to investigate *Plasmodium* liver stages in both cultured hepatocytes and in vivo. For instance, several HTL probes were optimized for standard and advanced imaging techniques, including super-resolution microscopy, enabling the exploration of UIS4 trafficking and turnover with unparalleled spatiotemporal resolution^50,52^. Moreover, the HaloTag system can be customized with activatable toxic or inhibitory molecules, like the photocaged MPS1 inhibitor reversine^53^ and photosensitizers^54^. Upon light absorption, photosensitizers generate reactive oxygen species (ROS) that can be harnessed for diverse biological applications, including the chromophore-assisted light inactivation of proteins, cell ablation, and in-situ deposition of imaging contrast agents^54^. With the transgenic parasites created in this study, photosensitizers could be employed to inactivate UIS4 during liver stage development and to investigate its subcellular distribution in combination with established electron microscopy techniques^55^.

This report highlights the intricate challenges involved in studying proximity-inducing therapeutics against novel classes of targets such as the PVM proteins of *Plasmodium* liver stages. Considering the ongoing difficulties in developing effective antimalarials against hypnozoites^3,6^, proximity-inducing therapeutics still constitute a promising approach that could make significant contributions to malaria eradication efforts. Beyond TPD, induced proximity could be utilized to modify the PVM molecular signature and trigger host cell-intrinsic immune responses promoting the clearance of *Plasmodium* liver stages^56^. More broadly, induced proximity holds potential as a novel therapeutic strategy against intracellular microbial pathogens, encompassing not only eukaryotic parasites but also bacteria and viruses^15^. It is anticipated that proximity-inducing approaches will address unmet medical needs and expand our therapeutic arsenal against latent and challenging infectious diseases.

## Methods

### Cell culture

The human hepatoma Huh7 cell line (Japanese Collection of Research Bioresources (JCRB) Cell Bank) was cultured in Dulbecco’s Modified Eagle Medium (DMEM) with high glucose (Gibco) supplemented with 10% heat-inactivated fetal bovine serum (FBS; Corning), 100 U/mL of penicillin-streptomycin (Gibco) and 1% GlutaMAX™ (Gibco) (hereafter referred to as cell culture media) at 37 °C and 5% CO_2_. Primary hepatocytes (BioIVT) were cultured in CP Medium (BioIVT, cat no. Z990003) supplemented with 1% PSN (Gibco, cat no. 15640055) (hereafter referred to as hepatocyte media) at 37 °C and 5% CO_2_, unless otherwise stated.

### Cell line generation

The following DNA plasmids and lentiviruses were provided by VectorBuilder: *P*_*CMV*_ -mCherry-GFP-HT (VB230409-1160fdt), *P*_*EF1a*_-HT-GFP-FIS1 (VB230406-1238ymp), *P*_*EF1a*_-HT-mCherry-FIS1 (VB240122-1674bkz), *P*_*UBC*_-SNAP tag-GFP (VB230607-1547nrb), *P*_*CMV*_-mCherry-VHL (VB230406-1232ghk), *P*_*CMV*_-mCherry-Ub (VB230406-1241ura), and *P*_*CMV*_-UIS4_*Pb*_-C_term_-HT-HiBiT (VB240122-1687zve). Briefly, Huh7 cells were seeded at a density of 3 × 10^5^ cells per well in a 6-well plate (Greiner) and transduced the next day with 3 × 10^5^ transduction units (TU) per well. Lentiviruses were added to cells in culture media containing 8 µg/mL polybrene, and cells were spun at 800 × g for 1 h at 37 °C, with low acceleration and break. One day post-transduction, lentiviruses were removed, and fresh cell culture media was added to cells. Transduced cells were then selected for 2–3 weeks in cell culture media containing 2 µg/mL puromycin (Gibco) or 7.5 µg/mL blasticidin (Gibco). The vector IDs listed above can be used to retrieve detailed information about the lentivirus vectors on vectorbuilder.com.

### Compounds

HaloPROTAC3 (Promega, cat no. GA3110), the fluorescent HaloTag ligand (Promega, cat no. G2801) and the bifunctional HaXS8 (Tocris, cat no. 4991) used in this study were commercially sourced. The VHL binder compound was produced internally at Novartis as previously described^20^. HaloPROTAC3, VHL binder and HaXS8 solutions were prepared in DMSO. DMSO-treated control conditions were included in all relevant experiments.

### Fluorescence activated cell sorting (FACS)

Huh7 cells that express fluorescent HT proteins were seeded at a density of 1.5 × 10^4^ cells per well in a 96-well flat bottom plate (Greiner) and treated with compounds for 24 h. Cells were detached and resuspended in 5 mM EDTA (Invitrogen) in the 96-well plate prior to measuring intracellular GFP and mCherry fluorescence using the BL1 and YL2 channels on the Attune flow cytometer (Thermo Fisher Scientific). The mean fluorescence of each sample was determined using the software FlowJo (v10.10) and expressed as percentages of DMSO-treated samples. For the mCherry-GFP-HT cells, similar fluorescence profiles were observed for mCherry and GFP, but only data for GFP fluorescence are presented.

### Nano-Glo HiBiT lytic assay

WT Huh7 and Huh7 UIS4_*Pb*_-C_term_-HT-HiBiT cells were seeded at a density of 8 × 10^3^ cells per well in a white 384-well plate (Greiner). One day post-seeding, cells were treated with compounds for 24 h before being assayed using the Nano-Glo HiBiT lytic kit (Promega). The luminescence of samples was measured on the CLARIOstar luminescent plate reader (BMG Labtech) 10 min after adding the reagents.

### Generation and characterization of *P. berghei* UIS4-HT

Transgenic *P. berghei* parasites expressing UIS4-HT were generated using a gene replacement strategy, as previously described with modifications^22^. Briefly, an insert DNA including the codon-optimized UIS4-HT fusion protein followed by the *P. berghei* DHFR/TS 3’ downstream region and the UIS4 flanking sequences was generated by DNA synthesis (Invitrogen) (see insert sequence, Supplementary Data S1). This insert DNA was then subcloned into the plasmid pL0005 (MRA-774, BEI Resources, contributed by Andrew P. Waters) using PCR (see primer sequences, Supplementary Table S1) and In-Fusion Cloning (Takara Bio). Positive colonies were identified using PCR analysis and the entire insert sequence of one clone was confirmed using DNA sequencing. The linearized UIS4-HT plasmid was introduced and integrated in *P. berghei* ANKA Cl15cy1 (MRA-871, BEI Resources, contributed by Chris J. Janse and Andrew P. Waters) parasites using previously described standard methods^57^ and passaged thrice in Swiss Webster mice with selection using pyrimethamine (PYR). Mice were then injected with 200 parasites (from the 3rd passage) and further treated with PYR. Infected blood was collected once parasitemia was > 1% and used to generate blood stocks and extract *P. berghei* genomic DNA. The genomic integration of UIS4-HT and the absence of contamination with WT parasites was confirmed using PCR analyses (Supplementary Fig. S1 and Supplementary Table S1).

### *P. berghei* liver stage infection assay

Huh7 cells were seeded at a density of 8 × 10^3^ cells per well in 384-well black plates (Greiner) one day prior to infection. *Anopheles stephensi* mosquitoes infected with *P. berghei* WT or *P. berghei* UIS4-HT were produced by the SporoCore (University of Georgia, UGA) and sporozoites were isolated by microdissection of salivary glands, as previously described with modifications^58,59^. Sporozoites were diluted in Roswell Park Memorial Institute 1640 medium (RPMI; Gibco) supplemented with 20% heat-inactivated FBS and Huh7 cells were then spun with 5 × 10^3^ sporozoites per well at 330 × g for 3 min, with low acceleration and break. Unless otherwise stated, infected cells were treated with compounds 2 h post-infection with media change. Infected cells were fixed using 4% paraformaldehyde (PFA; Electron Microscopy Sciences) 2 days post-infection and stored in DPBS at 4 °C until further processing.

### Staining and imaging in Huh7 cells

When required, the MitoTracker deep red probe (Invitrogen, cat no. M22426) was added to live cells and incubated for 45 min at 37 °C before fixation. Uninfected and infected fixed samples were permeabilized for 30 min at room temperature (RT) in blocking and permeabilization (BP) buffer containing 2% Bovine Serum Albumin (BSA) (Sigma-Aldrich) and 0.2% Triton X-100 (Sigma-Aldrich) in DPBS (Gibco). The samples were then incubated with the following for 3 h at RT in BP buffer: goat anti-*Pb* UIS4 (1:250, Origene, cat no. AB0042-200), rabbit anti-*Pc* HSP70^59^(1:5,000), mouse anti-HaloTag (1:1,000, Promega, cat no. G9211), rat anti-mCherry (1:100, Invitrogen, cat no. M11217) and/or mouse anti-GFP (1:200, Roche, cat no. 11814460001) antibodies, and/or a fluorescent HaloTag ligand (1:500, Promega G2801). The samples were washed thrice with DPBS and then stained with Hoechst (2 µg/mL, Thermo Fisher Scientific) and the following secondary antibodies (1:1,000) for 1 h at RT in BP buffer: AlexaFluor-488 donkey anti-Rabbit (Invitrogen, cat no. A32790), AlexaFluor-488 donkey anti-mouse (Invitrogen, cat no. A21202), AlexaFluor-568 donkey anti-goat (Invitrogen, cat no. A11057), AlexaFluor-568 donkey anti-rat (Invitrogen, cat no. A78946), AlexaFluor-568 donkey anti-rabbit (Invitrogen, cat no. A10042), AlexaFluor-647P donkey anti-rabbit (Invitrogen, cat no. A32795), AlexaFluor-647P donkey anti-mouse (Invitrogen, cat no. A32787) and/or AlexaFluor-647P donkey anti-goat (Invitrogen, cat no. A32849). The samples were then washed thrice with DPBS and stored in DPBS at 4 °C until imaging. Images were taken with a 20× objective and 25 fields of view were captured from each well of the 384-well plate on the ImageXpress Micro Confocal (IXMC; Molecular Devices) and processed using a Cell Profiler (v4.2.4) analysis pipeline for quantification. Representative pictures of cells and parasites were also taken using either the 20× or 40× objectives on the Laser Scanning Microscope 980 (LSM980) with Airyscan 2 (Zeiss) and processed with FIJI (v1.53t).

### *P. berghei* mouse infection model

Female C57BL/6 mice were injected retro-orbitally with 2.5 × 10^4^ *P. berghei* WT or UIS4-HT salivary glands sporozoites under isoflurane anesthesia. Blood samples were collected from the tail veins from 72 h post-infection and analyzed, unblinded, on slides using thin blood smears. The slides were then fixed in 100% methanol for 30s, followed by drying and staining with a 10% Giemsa solution for 15 min. After gentle washing with distilled water, the slides were dried and examined using a 100× oil immersion objective on the Nikon Eclipse E200 microscope. Parasitemia was calculated by determining the percentage of infected erythrocytes in 15–20 fields of view. Each field of view typically contained 100–200 total erythrocytes. Parasite DNA was extracted from the blood of sporozoite-infected mice and used to confirm the genotype of transgenic parasites.

### *P. cynomolgi* culture

*P. cynomolgi* Berok culture was performed as previously described^24,25^. Briefly, asexual parasite stages were propagated in rhesus erythrocytes (BioIVT) at a hematocrit of 3% in RPMI-1640 medium supplemented with GlutaMAX (Gibco), 30 mM HEPES (Sigma-Aldrich), 0.2% (w/v) of a 50% D-glucose solution, 200 µM hypoxanthine (Thermo Fisher Scientific), and 20% heat-inactivated *Macaca mulatta* serum (BioIVT). Parasite cultures were incubated at 37 °C in trimix gas (5% CO_2_, 5% O_2_, 90% N_2_).

### Preparation of the plasmid for the generation of *P. cynomolgi* UIS4-HT

The generation of transgenic *P. cynomolgi* Berok parasites was achieved as previously described with modifications^25^. Briefly, the gene editing of parasites was performed using a plasmid referred to as pCJ-110. pCJ-110 is based on the plasmid pYC-L2, designed for CRISPR/Cas9 editing of *P. yoelii*^60,61^. pYC-L2 was extensively remodeled with *P. cynomolgi* Berok-derived promoter and terminator sequences, amplified from Berok blood stage genomic DNA. Specifically, the resulting pCJ-110 encodes a cassette controlled by the bidirectional Berok EF1α promoter that drives the expression of mutant human dihydrofolate reductase (hDHFR) for positive selection and humanized SpCas9 for gene editing, separated by a 2A self-cleaving peptide. The bidirectional promoter also drives the expression of the bifunctional yeast fusion cytosine deaminase/uracil phosphoribosyltransferase (yFCU) for negative selection. The Berok HSP70 terminator is placed after the SpCas9 gene and the *P. falciparum* BiP terminator is placed after the yFCU gene. The plasmid also allows the expression of a single guide RNA (gRNA) from the Berok U6 promoter and includes a multiple cloning site facilitating donor template insertion required for the homology-directed double strand break repair. Thus, pCJ-110 allows for CRISPR/Cas9 transgenesis under both positive and negative selection. An illustration of the plasmid is shown in Supplementary Fig. S8. We amplified and sequenced the *uis4* locus (corresponding to PcM_0602100) from Berok blood stage gDNA with primers listed in Supplementary Table S1. The construction of the pCJ-110-UIS4-HT plasmid, which was used to tag the 3’ end of *P. cynomolgi* Berok *uis4* with the HaloTag (Supplementary Fig. S2) and to introduce silent shield mutations at the gRNA binding site, was performed in two steps. First, annealed oligonucleotides encoding the gRNA (Supplementary Table S1) were ligated into Esp3I (New England Biolabs, NEB)-digested pCJ-110 using T4 DNA ligase (NEB). Then, the plasmid was digested with SpeI and NotI (NEB) and reassembled with three PCR fragments encoding the 5’ and 3’ *uis4* homology regions (amplified from *P. cynomolgi* Berok genomic DNA) and the HaloTag sequence (amplified from the *P. berghei* UIS4-HT plasmid) (see primer sequences, Supplementary Table S1) using Gibson cloning (NEB). The sequence of the cloned donor template was confirmed using DNA sequencing.

### Generation of transgenic *P. cynomolgi* UIS4-HT

Transfection of *P. cynomolgi* was carried out using the pCJ-110-UIS4-HT plasmid as previously described with modifications^25,62^. Briefly, schizonts were purified with a Nycodenz gradient^23^ and a minimum of 1 × 10^7^ cells were resuspended in 100 µL of supplemented P3 primary cell solution (Lonza). Electroporation was performed using 20 µg of plasmid DNA and an Amaxa 4D electroporator (Lonza) as previously described^62^. To increase reinvasion efficiency, transfected parasites were transferred to 500 µL of pre-warmed culture medium at 20% hematocrit and incubated at 37 °C under shaking conditions for 1 h, prior to being further cultured using standard conditions. Transgenic parasites were selected with 50 nM PYR from 24 h post transfection, for 7 days. A transgenic population was obtained within 4 weeks after transfection and validated through PCR genotyping (Supplementary Fig. S2 and Supplementary Table S1). Blood stage UIS4-HT parasites were shipped to Oregon Health and Science University (OHSU) / Oregon National Primate Research Center (ONPRC) and stored as glycerolyte stocks in liquid nitrogen until use.

### *P. cynomolgi* infection of NHPs (OHSU / ONPRC)

Transgenic UIS4-HT parasites cycled in a Japanese macaque (JMac1, ~ 4.5-year-old male) and through the mosquito vector to a new Japanese macaque (JMac2, ~ 4.5-year-old female) were used to generate blood-stage glycerolyte stocks and to infect a naive Japanese macaque (JMac3, ~ 5-year-old female). Mosquitoes were then fed on the blood from this infected macaque and dissected to isolate salivary gland sporozoites. Sporozoites were counted, washed once in PBS and used to infect a rhesus macaque (~ 5-year-old male) intravenously (with 3.6 × 10^4^ sporozoites). Beginning 6 days post-infection, blood samples were collected for parasitemia quantification (Supplementary Fig. S2) and to monitor hematocrit and hematological factors associated with animal well-being. During peak infection, larger drawings of blood were collected to feed mosquitoes, generate glycerolyte stocks, and genotype parasites. At a maximum parasitemia of 3 × 10^5^ parasites/µL, NHPs were treated with Coartem tablets (20 mg artemether/120 mg lumefantrine, Novartis) twice daily for three days, which clear blood stage parasites but leave hypnozoites intact. Semi-weekly parasitemia monitoring was then conducted to detect relapse infections, arising from hypnozoite activation. Once a relapse was detected, monitoring of the macaque and mosquitoes feeding followed as described above. At the end of the study, the sporozoite-infected NHP was treated with a single dose of tafenoquine (Krintafel, 150 mg, GlaxoSmithKline), to clear any remaining hypnozoites, in addition to a course of Coartem. Blood stage transgenic parasite stocks obtained from a sporozoite-initiated infection were shipped to UGA and further used to produce infected mosquitoes.

### Infection of mosquitoes with *P. cynomolgi* (OHSU / ONPRC)

*Anopheles stephensi* mosquitoes were reared in-house as previously described^63^ or purchased from the SporoCore (UGA). Mosquitoes were maintained in a 26 °C incubator with 80% humidity and provided with sugar cubes and water pads until the day of infection. Briefly, blood was drawn from infected macaque into a Lithium-Heparin vacutainer (Greiner Bio-One). To prevent exflagellation of the male gametes prior to uptake by mosquitoes, the blood was maintained at 39 °C during transport from the non-human primate facility, through subsequent washing steps using pre-warmed media and during final transport to the insectary. RBCs were washed twice with RPMI and the serum volume was replaced with human AB^+^ sera. This was used to feed female mosquitoes aged 2–10 days using a glass membrane feeder covered with a single layer of parafilm. Infected mosquitoes were then provided a sugar cube and water pad daily and a supplemental blood meal via an anesthetized mouse at 4–6 days post-infection. Midgut oocysts were quantified in a sample of mosquitoes at 8–10 days post-infection and salivary gland sporozoites were isolated for downstream use at 16–19 days post-infection.

### *P. cynomolgi* infection of NHPs (UGA)

Cryopreserved *P. cynomolgi* UIS4-HT blood stocks were used to infect male Japanese macaques. After infection, parasitemia was monitored daily as previously described^64–66^. Parasitemia was monitored after inoculation through the last day of curative treatment using artemether-lumefantrine combination therapy.

### Infection of mosquitoes with *P. cynomolgi* (UGA)

*Anopheles dirus* mosquitoes were reared at UGA under controlled conditions of 27 °C and 80% relative humidity. Adult mosquitoes were maintained on 20% sucrose for the first three days after pupation and 10% sucrose for the remainder of development. Three- to five-day old female mosquitoes were used in membrane feeding experiments. Once parasitemia reached approximately 1 × 10^4^ parasites/µL, parasitized blood was collected from an infected animal into lithium heparin tubes and maintained at 37 °C throughout processing for feeding mosquitoes. Before membrane feeding, the blood was pelleted via centrifugation at 800 × g for 5 min, and the plasma was removed and discarded. The remaining pellet was resuspended in pre-warmed incomplete RPMI and centrifuged at 800 × g for 5 min. The supernatant was then removed and discarded, and the remaining pellet was resuspended at 50% hematocrit in pre-warmed, malaria-naïve Japanese macaque serum. The blood was placed in a glass-bell covered with parafilm membrane, and mosquitoes were allowed to feed for up to 30 min in the dark at 37 ^o^C. Afterwards, unfed mosquitoes were removed and discarded. Mosquitoes that engorged were maintained with 10% sucrose throughout the rest of the study. Six days after feeding, midguts were dissected from 5 to 10 mosquitoes and stained with 0.5% mercurochrome diluted with PBS (w/v). The prevalence of infected mosquitoes and the number of oocysts per midgut were quantified via light microscopy. Only feedings resulting in highly infected mosquitoes were used for liver stage studies.

### *P. cynomolgi* liver stage infection

Primary simian hepatocytes (BioIVT, lots HTV and HMP) were seeded at a density of 2.2 × 10^4^ cells per well in a collagen-coated 384-well black plate (Corning) two days prior to infection, as previously described^59^. Sporozoites were obtained from the salivary glands of mosquitoes infected with *P. cynomolgi* UIS4-HT (UGA) and collected in RPMI 1640 (KD Medical). Primary hepatocytes were infected with 1 × 10^4^ sporozoites per well in hepatocyte media, spun at 200 × g for 5 min and further incubated in hepatocyte media. Sporozoites were removed 24 h post-infection and hepatocyte media containing 5% PSN was added to infected cells. The 5% PSN hepatocyte media was changed on day four and six post-infection. The samples were fixed with 4% paraformaldehyde eight days post-infection and stored at 4°C in DPBS until processed.

### Immunofluorescence assay with primary hepatocytes

Fixed samples were incubated for 1 h at RT in BP buffer. The samples were then incubated at 4 ^o^C overnight in BP buffer containing a combination of either rabbit anti-*Pc* HSP70^59^ (1:5,000) and mouse anti-HaloTag (1:1,000, Promega, cat no. G9211), or rat anti-mCherry (1:200, Invitrogen, cat no. M11217) and mouse anti-GFP (1:100, Roche, cat no. 11814460001) primary antibodies. The next day, the samples were washed thrice with DPBS and stained with Hoechst (2 µg/mL, Invitrogen), AlexaFluor-568 goat anti-rabbit (Invitrogen, cat. A11036), AlexaFluor-488 goat anti-mouse (Invitrogen, cat. A11001) and/or AlexaFluor-568 goat anti-rat (Invitrogen, cat. A11077) (1:1,000) for 2 h at RT in BP buffer. The samples were then washed thrice with DPBS and stored in DPBS at 4 °C until imaging on the LSM 980 equipped with Airysan 2 (Zeiss) or the IXMC (Molecular Devices).

### HaloPROTAC3-mediated degradation assay in primary human hepatocytes

Primary human hepatocytes (BioIVT, lots QWK and BGW) were seeded at a density of 2 × 10^4^ cells per well in a collagen-coated 384-well black plate (Corning) and transduced with 1 × 10^5^ TU of the *P*_*CMV*_-mCherry-T2A-EGFP-HT-HiBiT lentivirus (Vector Builder, VB230405-1468zfc) 48 h post-cell seeding. The construct delivered by this lentivirus allows the host cell to express both a transduction marker (i.e., mCherry) and a degradation reporter (i.e., EGFP-HT-HiBiT), separated by the T2A self-splicing peptide. Briefly, lentiviruses were added to cells in hepatocyte media supplemented with 8 µg/mL of polybrene, and cells were spun at 800 × g for 1 h at 37 ^o^C. The next day, lentiviruses were removed, and fresh hepatocyte media was added to the cells. Two days later, transduced cells were treated with compounds for 48 h and then fixed with 4% paraformaldehyde for 30 min at RT, immunostained, and imaged. To evaluate the degradation of EGFP-HT-HiBiT following HaloPROTAC3 treatment, 9 fields of view were imaged per technical duplicate using the 20× objective of the IXMC and analyzed using a Cell Profiler pipeline. Briefly, AlexaFluor488 (immunostained GFP) and AlexaFluor568 (immunostained mCherry) fluorescence intensities were measured in the area surrounding nuclei. Transduced cells were defined as cells with mCherry intensities greater than three standard deviations from the background intensity measured in non-transduced cells. Then, ratios of mean intensities associated with the GFP and mCherry markers were evaluated. We defined 0% GFP expression as the ratio of GFP background in non-transduced cells to the mCherry signal in transduced, DMSO-treated cells. The GFP/mCherry ratio in transduced, DMSO-treated cells was defined as 100% GFP expression.

### Preparation of graphs, statistical analysis, and artworks

Using an image-processing Cell Profiler pipeline, number of liver stages, number of host nuclei, fluorescence intensities, and other parameters were quantified before analysis on TIBCO Spotfire (1.0_L_EN_02) and GraphPad Prism (version 9.5). The data generated was then used to create bar charts and response curves. Dose response data were normalized (100%, DMSO-treated samples) and plotted with the curve fit setting “log(inhibitor) vs. normalized response -- Variable slope” on Prism. Specific statistical tests, number of technical replicates and number of independent experiments (N) are indicated in figure legends. Prism, FIJI (v1.53t), BioRender (biorender.com), Adobe Illustrator (v28.3), SnapGene® software (from Dotmatics; available at snapgene.com) and ChemDraw (revvity, Signals ChemDraw, v23.1.1.3) were used to create figures. Some micrographs were pseudocolored for representation.

## Electronic supplementary material

Below is the link to the electronic supplementary material.

Supplementary Material 1

Supplementary Material 2

## Data Availability

Requests for compounds and transgenic cell lines and parasites are subject to Material Transfer Agreements (Global Health Disease Area, Biomedical Research, Novartis). All data are available from the corresponding authors upon reasonable request.
