# Supplementary material for: HaloPROTAC3 does not trigger the degradation of the halotagged parasitophorous vacuole membrane protein UIS4 during Plasmodium liver stage development
Source: Sci Rep. 2025 May 26;15:18323. doi: 10.1038/s41598-025-98257-9 (PMC12106710; doi:10.1038/s41598-025-98257-9)
Supplement: Supplementary file 1 — Supplementary Material 1 [file 41598_2025_98257_MOESM1_ESM.docx]

**HaloPROTAC3 does not trigger the degradation of the HaloTagged parasitophorous vacuole membrane protein UIS4 during *Plasmodium* liver stage development**

Supplementary Information

Melanie Lam^1^, Alexandra Probst^2^, Laura Torres^1^, Ashley A. Lantigua^1^, Matthew E. Fishbaugher^2^, Jyothsna R. Kumar^2^, Manuel Saldivia^2^, Allison Torres^2^, Shreeya Hegde^2^, Maya Aleshnick^3^, Charlie Jennison^4^, Sarah G.H. Roberson^5^, Chester J. Joyner^5^, Ashley M. Vaughan^4^, Brandon K. Wilder^3^, Carole Manneville^6^, Erika L. Flannery^2^, David Marcellin^6^, Beat Nyfeler^6^, Zacharias Thiel^6^, Sebastian A. Mikolajczak^2^, Anke Harupa^2*^, Gabriel Mitchell^1*^

^1^ Open Innovation at Global Health Disease Area, Biomedical Research, Novartis, Emeryville, CA, USA

^2^ Global Health Disease Area, Biomedical Research, Novartis, Emeryville, CA, USA

^3^ Oregon Health and Science University, Vaccine and Gene Therapy Institute, Beaverton, Oregon, USA

^4^ Seattle Children’s Research Institute, Seattle, Washington, USA

^5^ Department of Infectious Diseases, University of Georgia, Athens, Georgia, USA

^6^ Discovery Sciences, Biomedical Research, Novartis, Basel, Switzerland

*Corresponding authors: [anke.harupa-chung@novartis.com](mailto:anke.harupa-chung@novartis.com) and [gabrielmitchell2@gmail.com](mailto:gabrielmitchell2@gmail.com)

**
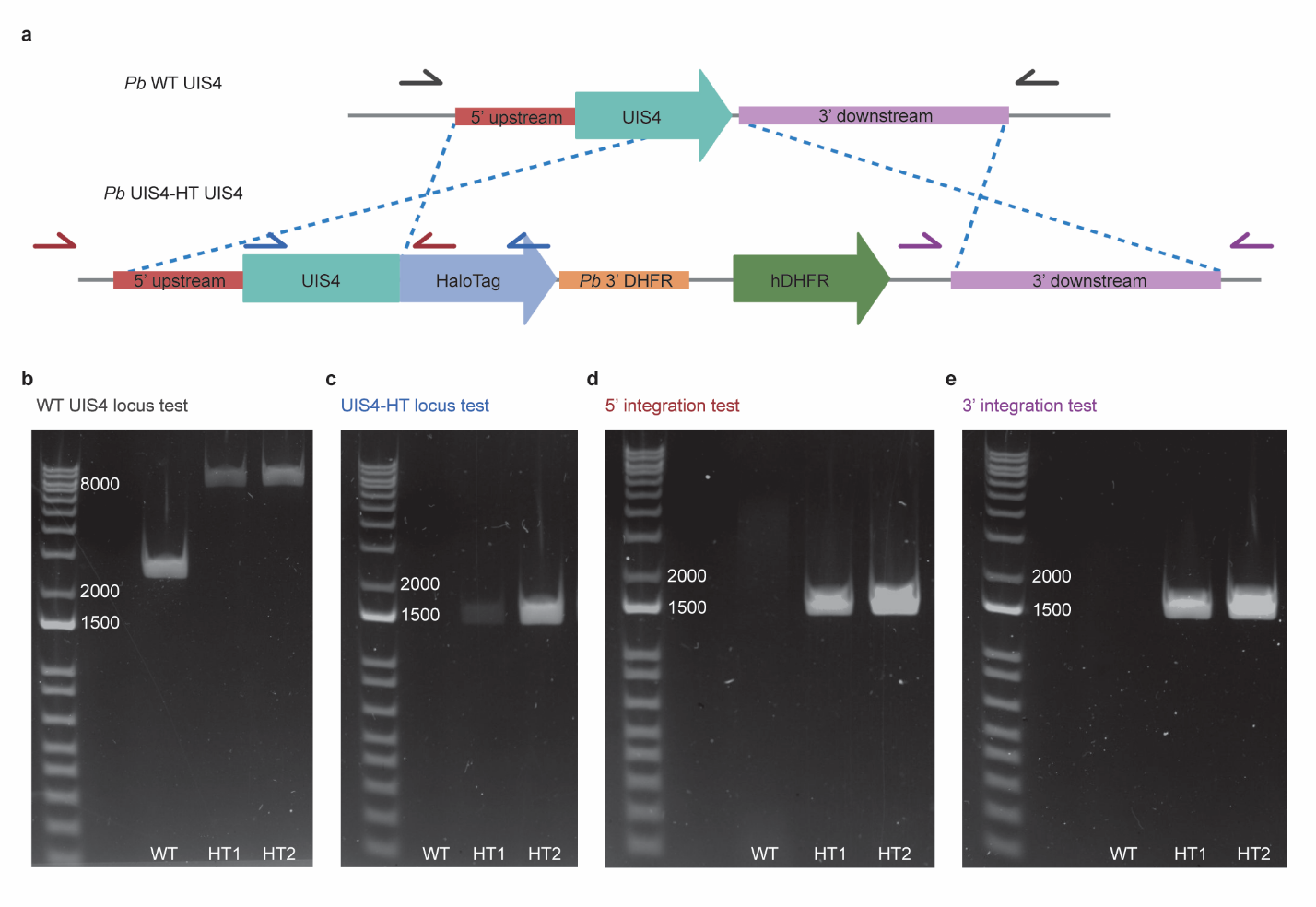
**

**Supplementary Figure S1.** Genotyping of *P. berghei* (*Pb*) UIS4-HT. **(a)** Schematic of the strategy used to generate and genotype *P. berghei* UIS4-HT. The gray arrows represent a primer set that amplifies the WT UIS4 locus (~2,500bp). This primer set also amplifies a longer fragment, corresponding to the transgenic UIS4-HT locus (~8,000bp). The blue set of arrows are primers designed to amplify a fragment specific to the UIS4-HT fusion (~1,500bp), and the red and purple arrows are primers designed to amplify fragments specific to the 5’ and 3’ junctions (~1,500bp) found in *P. berghei* UIS4-HT, respectively. **(b-e)** Representative DNA gels are shown for each test and for *P. berghei* WT and UIS4-HT parasites. HT1 and HT2 represents different *P. berghei* UIS4-HT isolates (only HT2 was used in this study). The DNA E-Gel™ 1 Kb Plus DNA Ladder (Invitrogen) is displayed.


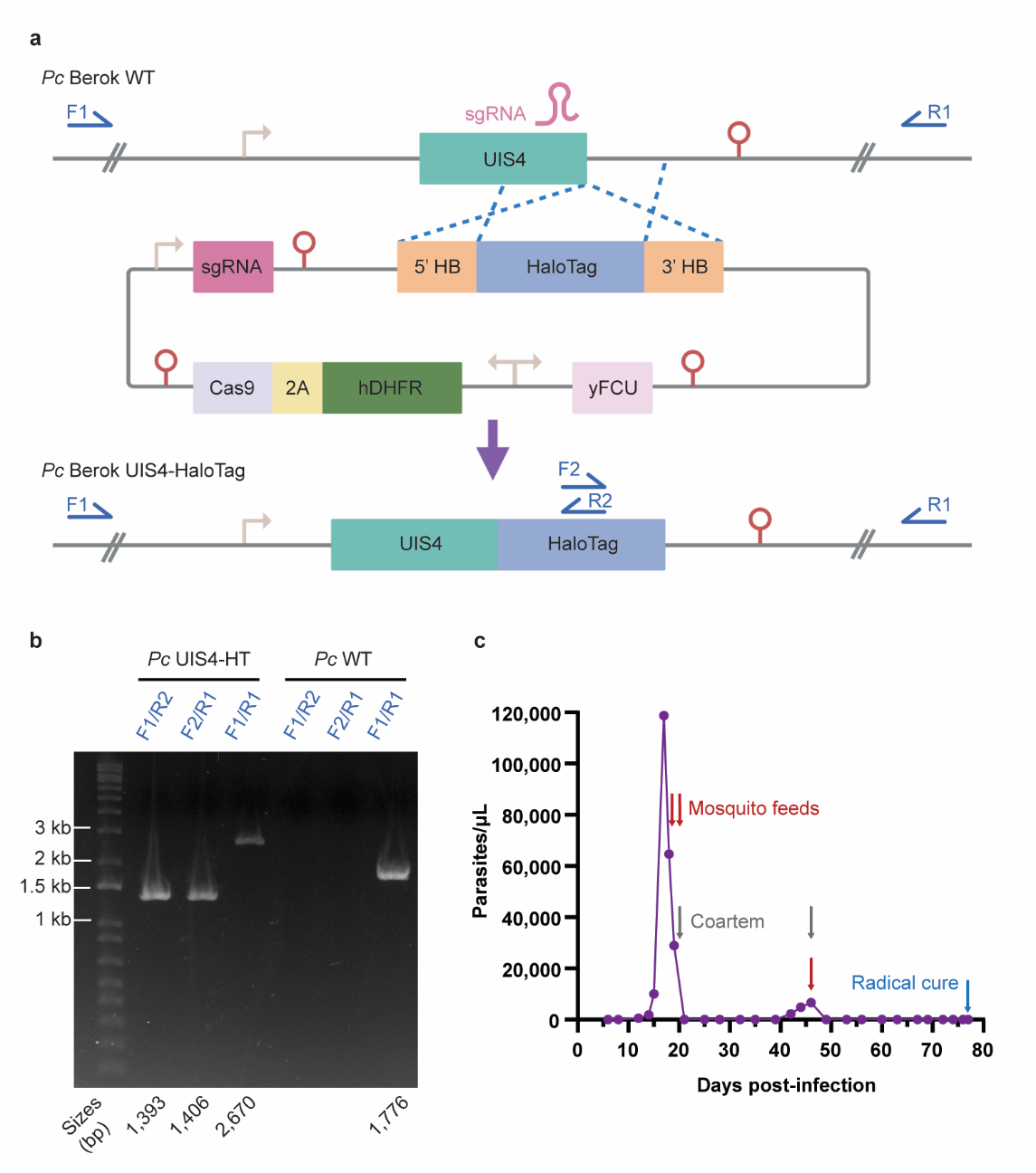


**Supplementary Figure S2.** Genotyping of *P. cynomolgi* (*Pc*) UIS4-HT. **(a)** Schematic of the strategy used to generate and genotype *Pc* Berok UIS4-HaloTag. The WT and UIS4-HT loci as well as the plasmid used for CRISPR/Cas9 genome editing are shown. The single guide RNA targeted the 3’ end of *uis4*, as indicated. The PCR primers (i.e., F1, R1, F2 and R2) used for genotyping are indicated. **(b)** PCR genotyping of *Pc* UIS4-HaloTag confirmed editing of the locus. PCR reactions using *Pc* WT genomic DNA served as controls. The DNA E-Gel™ 1 Kb Plus DNA Ladder (Invitrogen) is displayed and expected band sizes are indicated at the bottom for each PCR reaction. **(c)** In vivo blood stage parasitemia in a rhesus monkey (~5-year-old male) infected with 36,000 *Pc* UIS4-HT sporozoites. Blood stage parasites were detected 12 days post-infection and a relapse was observed 42 days post-infection. Arrows indicate mosquito feeds (red), the start of 3-day drug treatments with Coartem (grey) and the start of the final radical cure drug treatment with Coartem (3-day) and tafenoquine (single-dose) (blue). HB, homology box; 2A, 2A self-cleaving peptide; hDHFR, human dihydrofolate reductase; yFCU, bifunctional yeast fusion cytosine deaminase/uracil phosphoribosyltransferase.

**
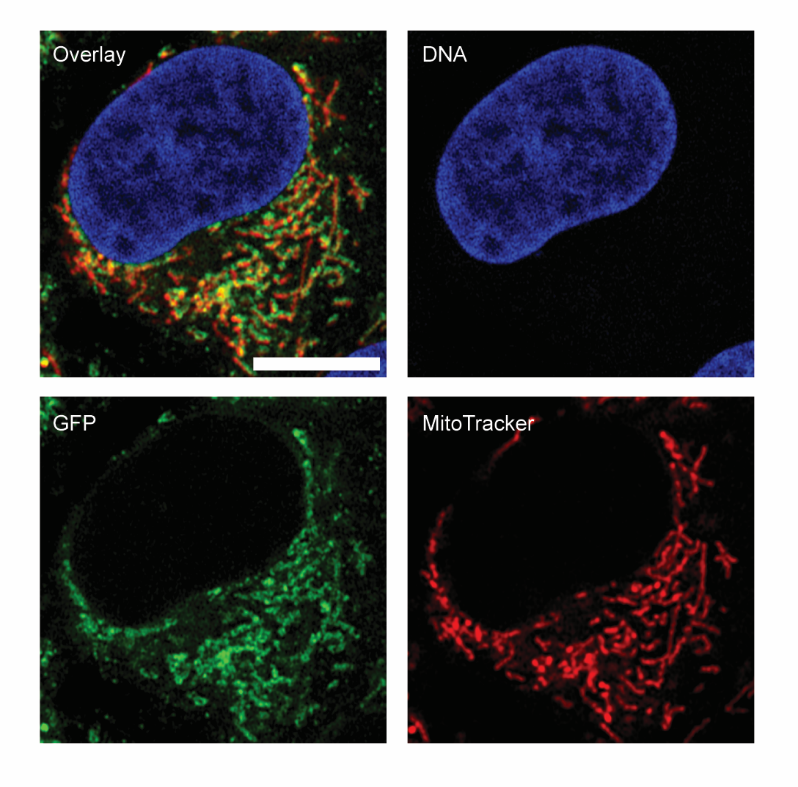
**

**Supplementary Figure S3.** Airyscan images of Huh7 HT-GFP-FIS1 cells. Airyscan micrographs of fixed Huh7 HT-GFP-FIS1 cells stained for DNA (blue) and MitoTracker (red). The endogenous GFP fluorescence (green) is also shown. Scale bar is 10 µm. Note the colocalization of HT-GFP-FIS1 with Mitotracker.

**
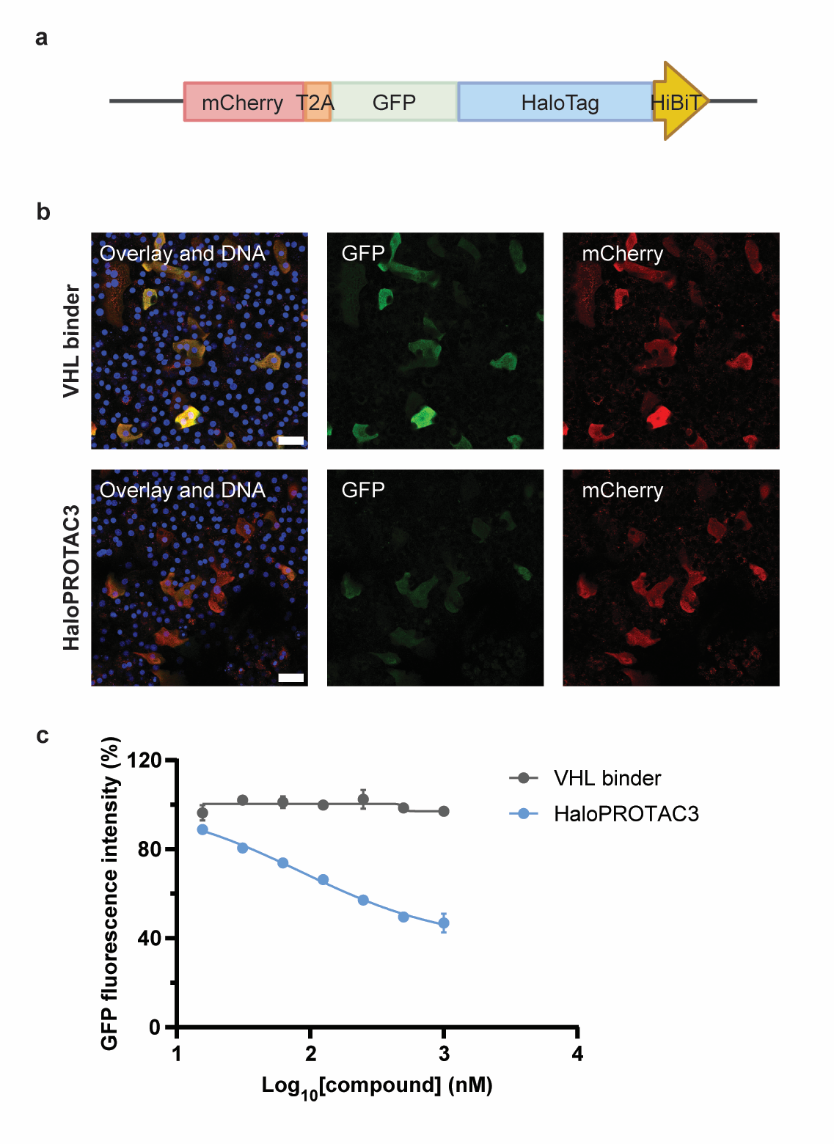
**

**Supplementary Figure S4.** HaloPROTAC3 triggers degradation of a soluble protein in primary human hepatocytes. **(a)** Illustration of the mCherry-T2A-GFP-HT-HiBiT lentiviral construct used to transduce primary human hepatocytes. **(b)** Confocal micrographs of transduced primary human hepatocytes treated with 1 µM VHL binder or HaloPROTAC3. DNA (blue), GFP (green), and mCherry (red) are shown. Scale bars are 50 µm. **(c)** Dose-response curves showing that HaloPROTAC3 triggers degradation of the GFP-HT-HiBiT fusion protein in primary human hepatocytes. 100% corresponds to the GFP/mCherry ratio in transduced, DMSO-treated cells. Results are expressed as means with SEMs (N=2).

**
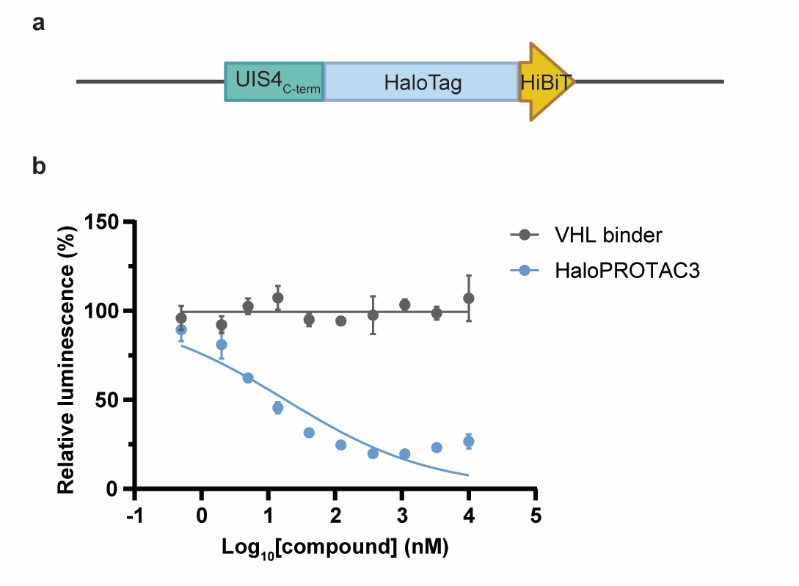
**

**Supplementary Figure S5.** HaloPROTAC3 triggers the degradation of the C-terminal domain of *P. berghei* (*Pb*) UIS4. **(a)** Illustration of the *Pb* UIS4_C-term_-HaloTag-HiBiT construct used to perform a degradation assay based on a luminescence readout. **(b)** Dose-response curves showing that HaloPROTAC3 triggers the degradation of *Pb* UIS4_C-term_-HaloTag-HiBiT in Huh7 cells. Luminescence values were normalized and averaged from technical duplicates. 100% corresponds to DMSO-treated samples. Results are means with SEMs (N=2).

**
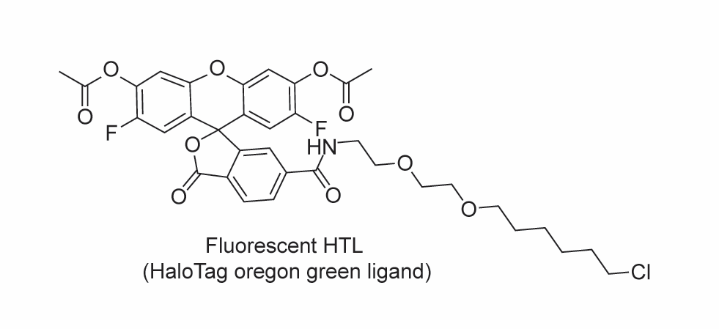
**

**Supplementary Figure S6.** Chemical structure of the fluorescent HaloTag ligand used in this study.

**
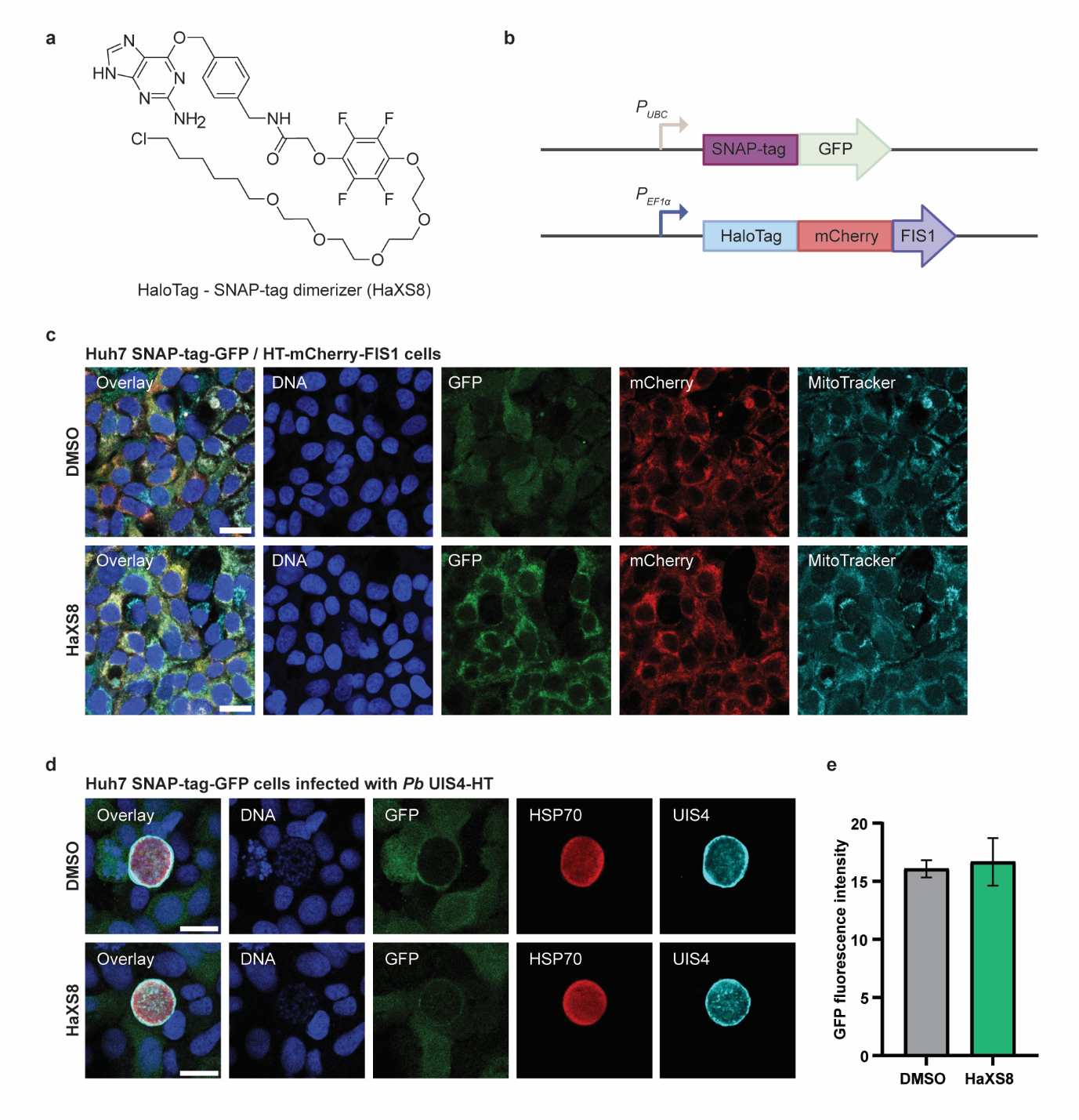
**

**Supplementary Figure S7.** HaXS8 triggers the recruitment of SNAP-tag-GFP to host HT mitochondria, but not to *P. berghei* (*Pb*) UIS4-HT. **(a)** Chemical structure of the heterofunctional compound HaXS8, a HaloTag - SNAP-tag dimerizer. **(b)** Illustration showing the SNAP-tag-GFP and the HaloTag-mCherry-FIS1 constructs expressed in Huh7. **(c)** Confocal micrographs showing the Huh7 SNAP-GFP / HT-mCherry-FIS1 cells treated with DMSO or 13.7 nM HaXS8 and stained for DNA (blue), GFP (green), mCherry (red), and MitoTracker (cyan). The HaXS8 treatment resulted in colocalization of GFP with the mitochondria. Scale bars are 20 µm. **(d)** Confocal micrographs of Huh7 SNAP-tag-GFP cells infected with *Pb* UIS4-HT, treated with DMSO or 13.7 nM HaXS8, and stained for DNA (blue), GFP (green), and parasite markers HSP70 (red) and UIS4 (cyan). Scale bars are 20 µm. **(e)** Integrated GFP intensities associated with *Pb* UIS4-HT liver stages in Huh7 cells treated with DMSO or 13.7 nM HaXS8. Median values were determined for each well and averaged from technical duplicates. Results are not statistically significantly different (*P*=0.81, unpaired *t* test) and are expressed as means with SEMs (N=2).

**
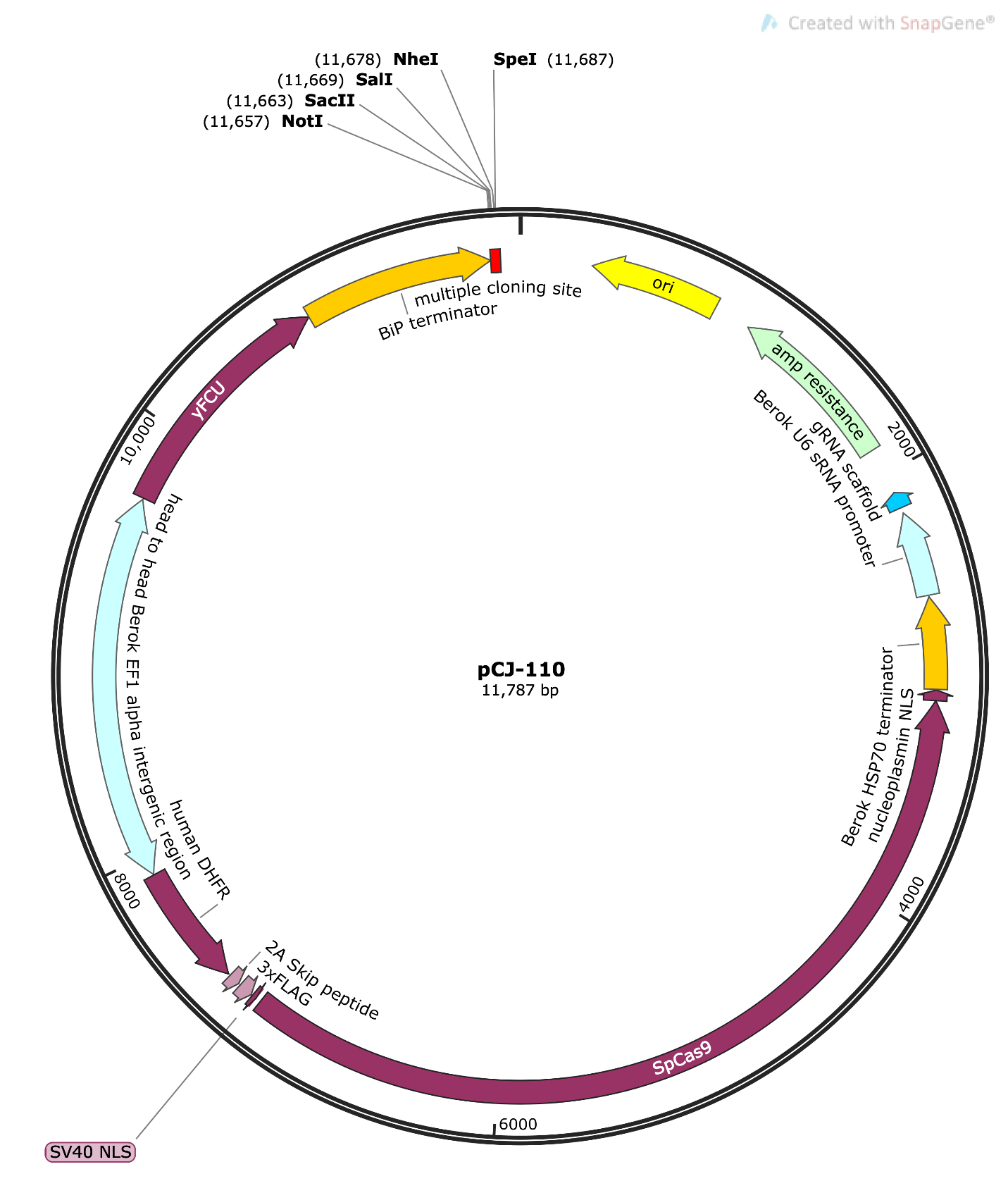
**

**Supplementary Figure S8.** Map of plasmid pCJ-110. Ori, origin of replication; amp resistance, ampicillin resistance; HSP70, heat shock protein 70; NLS, nuclear localization signal; SV40, simian virus 40; 2A skip peptide or 2A self-cleaving peptide; DHFR, dihydrofolate reductase; yFCU, bifunctional yeast fusion cytosine deaminase/uracil phosphoribosyltransferase; BiP, binding immunoglobulin protein.

**Supplementary Table S1.** Primers used in this study^a^

| Description | | Primer # | Sequence (5’-3’) |
| --- | --- | --- | --- |
| *P. berghei* | |  |  |
| Plasmid preparation | |  |  |
|  | Linearization pL0005 (Fwd) | P-Pb-01 | TCACCGTCATCACCGAAA |
|  | Linearization pL0005 (Rev) | P-Pb-02 | AAACCTCTGACACATGCA |
|  | UIS4-HT insert (Fwd) | P-Pb-03 | GCTGCATGTGTCAGAGGTTT |
|  | UIS4-HT insert (Rev) | P-Pb-04 | CGTTTCGGTGATGACGGTGA |
| Genotyping | |  |  |
|  | 5’ integration test (Fwd) | P-Pb-05 | AATTTGTAATTTATTCGAGGGTATCA |
|  | 5’ integration test (Rev) | P-Pb-06 | AAACCTGTTCCTATTTCTGCCA |
|  | 3’ integration test (Fwd) | P-Pb-07 | GCACTCTCAGTACAATCTGC |
|  | 3’ integration test (Rev)^b^ | P-Pb-08 | AGCAGCTAATGTCAATATATTTTATG |
|  | UIS4 locus test (Fwd) | P-Pb-09 | TTATGGTTGATCCTTTCCTTTTATG |
|  | UIS4 locus test (Rev)^b^ | P-Pb-08 | AGCAGCTAATGTCAATATATTTTATG |
|  | UIS4-HT test (Fwd) | P-Pb-10 | CCACATACGTTTCTCTATTTTT |
|  | UIS4-HT test (Rev) | P-Pb-11 | CTAAAGTACTTAACCATCTTGCT |
| *P. cynomolgi* | |  |  |
| Sequencing of *Pc* Berok UIS4 locus | |  |  |
|  | Amplification UIS4 locus (Fwd) | P-Pc-01 | CTTAGTGGTCTTGCGATCGTC |
|  | Amplification UIS4 locus (Rev) | P-Pc-02 | GCAGGCCACAGGATTCAAC |
|  | Sequencing 1 (Fwd) | P-Pc-03 | CAACGGTTGAAGGAACACCTC |
|  | Sequencing 2 (Rev) | P-Pc-04 | GGGTAAAATGGCGAAAAGGG |
|  | Sequencing 3 (Rev) | P-Pc-05 | CCATTATCCACACAGGTACGG |
|  | Sequencing 4 (Fwd) | P-Pc-06 | GAGATTGGCCAAGAAGCAGG |
| Plasmid preparation | |  |  |
|  | Annealing of gRNA (Fwd) | P-Pc-07 | TATTGGTGATACAGGTGAGAAATT |
|  | Annealing of gRNA (Rev) | P-Pc-08 | AAACAATTTCTCACCTGTATCACC |
|  | 5’ HB insert (Fwd)^c^ | P-Pc-09 | CATGATTACGCCAAGCTTATACTAGTGCAGCTGTTTTGCATTCTCT |
|  | 5’ HB insert (Rev)^c,d^ | P-Pc-010 | GGTCGAAAAAAGAGTCTCCTAACTTTTCTCCAGTGTCTCCGTCTACTCCCTCGATGACGACATTGGGTTCG |
|  | 3’ HB insert (Fwd)^c^ | P-Pc-011 | AAGTACTTTAGAAATTTCTGGATAAAGCGTACTGTCTACGCCAC |
|  | 3’ HB insert (Rev)^c^ | P-Pc-012 | GTTATATAGCAAAAGAAAAGAAAGCGGCCGCGTCAAACGGGGAAAATGCAC |
|  | HaloTag insert (Fwd)^c,d^ | P-Pc-13 | GGAGACTCTTTTTTCGACCAACCTTTTAACGTAAACGTAACAAATTCACCAATAATTATGGCAGAAATAGGAACAGG |
|  | HaloTag insert (Rev)^c^ | P-Pc-14 | TTATCCAGAAATTTCTAAAGTACTTAACC |
| Genotyping | |  |  |
|  | UIS4 locus test (F1) (Fwd)^b,e^ | P-Pc-15 | ACAACTTCGCTCGTTTTCC |
|  | UIS4 locus test (R1) (Rev)^b,e^ | P-Pc-16 | TGTGCCCACCTATATATCTG |
|  | 5’ integration test (F1) (Fwd)^b,e^ | P-Pc-15 | ACAACTTCGCTCGTTTTCC |
|  | 5’ integration test (R2) (Rev)^e^ | P-Pc-17 | ACATCAGTTGTTCTAAAAGCT |
|  | 3’ integration test (F2) (Fwd)^e^ | P-Pc-18 | TTGGGCAAAAAGAAATCCTG |
|  | 3’ integration test (R1) (Rev)^b,e^ | P-Pc-16 | TGTGCCCACCTATATATCTG |

^a^ Fwd, forward; Rev, reverse; HB, homology box; Pb, *P. berghei*; Pc, *P. cynomolgi*.

^b^ These primers were used for more than one PCR reaction.

^c^ Include overhangs for Gibson assembly.

^d^ Include silent mutations.

^e^ F1, R1, F2 and R2 refers to the primer labels used in Supplementary Figure S2.
